# Supplementary material for: SPEAKS study: study protocol of a multisite feasibility trial of the Specialist Psychotherapy with Emotion for Anorexia in Kent and Sussex (SPEAKS) intervention for outpatients with anorexia nervosa or otherwise specified feeding and eating disorders, anorexia nervosa type
Source: BMJ Open. 2022 Feb 22;12(2):e050350. doi: 10.1136/bmjopen-2021-050350 (PMC8867350; doi:10.1136/bmjopen-2021-050350)
Supplement: Author's manuscript [file bmjopen-2021-050350.draft_revisions.pdf]

BMJ Open is committed to open peer review. As part of this commitment we make the peer review history of every article we publish publicly available.

When an article is published we post the peer reviewers' comments and the authors' responses online. We also post the versions of the paper that were used during peer review. These are the versions that the peer review comments apply to.

The versions of the paper that follow are the versions that were submitted during the peer review process. They are not the versions of record or the final published versions. They should not be cited or distributed as the published version of this manuscript.

BMJ Open is an open access journal and the full, final, typeset and author-corrected version of record of the manuscript is available on our site with no access controls, subscription charges or pay-per-view fees (http://bmjopen.bmj.com).

If you have any questions on BMJ Open's open peer review process please email info.bmjopen@bmj.com

BMJ Open

The SPEAKS study: Study protocol of a multisite feasibility trial of the Specialist Psychotherapy with Emotion for Anorexia in Kent and Sussex (SPEAKS) intervention for outpatients with anorexia nervosa or otherwise specified feeding and eating disorders, anorexia nervosa type

Journal:	*BMJ Open*
Manuscript ID	bmjopen-2021-050350
Article Type:	Protocol
Date Submitted by the Author:	19-Feb-2021

Complete List of Authors:	Oldershaw, Anna; North East London NHS Foundation Trust Goodmayes Hospital, Kent and Medway All Age Eating Disorder Service; Canterbury Christ Church University Faculty of Social and Applied Sciences, Salomons Institute for Applied Psychology<br>Lavender, Tony; Canterbury Christ Church University, Salomons Institute for Applied Psychology<br>Basra, Randeep; North East London NHS Foundation Trust Goodmayes Hospital, Kent and Medway All Age Eating Disorder Service<br>Startup, Helen; Sussex Partnership NHS Foundation Trust, Brighton and Hove Eating Disorder Service

Keywords:	Eating disorders < PSYCHIATRY, Adult psychiatry < PSYCHIATRY, QUALITATIVE RESEARCH

SCHOLARONE™
Manuscripts

The SPEAKS study: Study protocol of a multisite feasibility trial of the Specialist Psychotherapy with Emotion for Anorexia in Kent and Sussex (SPEAKS) intervention for outpatients with anorexia nervosa or otherwise specified feeding and eating disorders, anorexia nervosa type

Anna Oldershaw, Tony Lavender, Randeep Basra & Helen Startup

For peer review only

For peer review only - http://bmjopen.bmj.com/site/about/guidelines.xhtml

Abstract

Introduction

Anorexia nervosa (AN) is a severe mental health condition associated with high levels of mortality and impairment and significantly impaired quality of life. National guidelines outline psychotherapeutic interventions as the treatment of choice for adults with AN, but outcomes limited and therapy drop-out is high, resulting in calls for new innovative treatments. The Specialist Psychotherapy with Emotion for Anorexia in Kent and Sussex (SPEAKS) research programme sought to develop the SPEAKS intervention avoiding some of the difficulties inherent in development of earlier interventions, such as lack of clarity of change processes. The intervention focuses on a core hypothesized maintaining factor (emotional experience) with clear proposed model of change. The current feasibility trial aims to provide an initial test of the SPEAKS intervention.

Methods & Analysis

Up to 60 participants (36 therapy completers) meeting inclusion criteria will be offered the SPEAKS intervention instead of treatment-as-usual (TAU). SPEAKS is a weekly psychotherapy lasting nine to 12 months, provided by trained and experienced eating disorders therapists. All other clinical input remains inline with TAU. *Validity and acceptability* will be assessed using VAS scales and end of therapy interview. *Reach and recruitment*, such as conversion rate, will be monitored. To support *sample size estimation* and *economic estimation*, data pertaining to eating disorder-related symptoms will be recorded every three months, alongside service usage and intervention specific measures. Videoed therapy sessions will inform model adherence. Additional analyses coding videoed therapy sessions will test SPEAKS change process hypotheses.

Ethic & Dissemination

Ethical approval has been granted by the London–Bromley Research Ethics Committee (NHS Rec Reference: 19/LO/1530). Data will be disseminated via high-impact, peer-reviewed journals (using

For peer review only - http://bmjopen.bmj.com/site/about/guidelines.xhtml

Open Access where possible), conferences, service user and charity networks (e.g. BEAT) and through a free open conference hosted by the NHS Trusts and HEI.

Article summary: Strengths and Limitations

- This feasibility trial studies a novel psychotherapy for adults with AN, with a focus on emotional change: The SPEAKS intervention.
- This feasibility study is strengthened by its multi-site trial design, completed in largely 'research naïve' NHS services, increasing insight into its feasibility and generalisability.
- In addition to its feasibility aims regarding the SPEAKS intervention, the trial design includes analysis of the therapeutic change process with wider implications for understanding therapeutic change for adults with AN.
- The trial has been subject to the challenges imposed by the COVID-19 pandemic including regarding clinical and research engagement; however it has achieved all recruitment targets.

Keywords: Anorexia nervosa, eating disorders, emotions, emotion focused therapy, schema therapy

For peer review only - http://bmjopen.bmj.com/site/about/guidelines.xhtml

Trial registration

Registered on ISRCTN trials registry reference ISRCTN11778891. Prospectively applied 28th November 2019; retrospectively assigned 20th January 2020.

http://www.isrctn.com/ISRCTN11778891

Trial Status

Recruitment began on 12th December 2019 and ends 28th February 2021. All data will be collected and the trial ended by 28th February 2022.

Protocol version

SPEAKS protocol version 3.0 (30/08/2020).

Trial sponsor information

North East London NHS Foundation Trust

Research and Development Department

1st Floor, Maggie Lilley Suite

Goodmayes Hospital

Barley Lane

Ilford

IG3 8XJ

+44 (0)300 555 1200

Fiona.Horton@NELFT.nhs.uk

Word Count: 3,807

For peer review only - http://bmjopen.bmj.com/site/about/guidelines.xhtml

Introduction

Background and rationale

Anorexia nervosa (AN) is a severe mental illness with poor prognosis and the highest mortality rate of any psychiatric disorder. Early intervention is key, but is impeded by a delay of around 18 months from symptoms emerging to treatment, and with multiple relapses likely[1]. Caregiving for somebody with severe AN is almost twice as lengthy as for somebody with a physical health disorder (e.g., cancer) or other mental health difficulty (e.g., psychosis)[2]. Thus significant costs exist financially and emotionally to the individual, family and carers, as well as society as a whole, offering a “compelling case for change” in services and treatment[1, p. 9].

Most recent NICE guidelines in the UK recommend that psychotherapeutic interventions be considered for adults with AN[3]. Yet results from randomized controlled trials (RCTs) indicate that out-patient treatments do not out-perform each other or control comparisons[4-8] with weight-restoration achieved by 20 percent of patients after one year[6-8]. AN is highly valued by sufferers resulting in poor treatment engagement and high dropout rates[9]. Thus there is an urgent need to develop innovative interventions that can engage people with AN[3, 10, 11], while targeting unique factors involved in its development and maintenance[12]. Emotional experience has long been recognised as a factor in the development and maintenance of AN and is recognised as a promising target area for therapies[13] and increasingly incorporated into psychotherapy interventions for adults with AN[4-8]. However, outcomes remain limited and it is unclear to what extent emotional difficulties are targeted by these interventions[14].

This trial comprises part of the SPEAKS programme which aimed to develop and test in a feasibility study an emotion focused intervention for adults with anorexia (the SPEAKS intervention). This research programme sought to overcome several key difficulties with the development and application of some earlier interventions, such as unsuccessful targeting of variables or lack of clarity

For peer review only - http://bmjopen.bmj.com/site/about/guidelines.xhtml

on how change is achieved. The SPEAKS programme proposes focussing on a core clearly defined model with one key putative maintaining process (emotional experience in AN) and following an 'interventionist-causal model approach'[15]. By developing a new model of the development and maintenance of AN drawn from integration of quantitative and qualitative research, it seeks to ensure an emotions' focus[16]. The SPEAKS intervention hypothesises a clear and testable change process to be targeted in therapy[17]; thus affording the ability to examine proposed mechanisms of change enabling further evidence-based development and refinement of the model. This body of work involved close partnering with stakeholders including those with current and past experience of AN, families, therapists and service managers.

Objectives

This multi-site feasibility trial aims to investigate the SPEAKS intervention in the following domains:

- Validity and acceptability
- Reach and recruitment
- Adherence and compliance
- Sample size & economic evaluation
- Change process analysis

We hypothesise that:

(1) SPEAKS will be acceptable to participants and therapists.

(2) SPEAKS will meet sufficient reach and recruitment expectations to support progression to full RCT.

(3) Therapists and service users will be able to adhere to the therapy model and research requirements

(4) The SPEAKS intervention will follow our hypothesised change process

For peer review only

Trial design

The SPEAKS study is a multi-site single armed within-group mixed methods design. The study design is displayed in Figure 1.

For peer review only

For peer review only - http://bmjopen.bmj.com/site/about/guidelines.xhtml

Methods: Participants, interventions and outcomes

Study setting

This feasibility study runs in two outpatient specialist eating disorders service within the UK National Health Service (NHS): Kent & Medway All Age Eating Disorder Service at NELFT and Sussex Eating Disorder Service at SPFT.

Eligibility criteria

Service Users

Service users are eligible to participate if they:

(1) Are referred into the All Age Eating Disorder Service in Kent or Sussex Eating Disorder Service and meet service criteria (i.e. they are registered with a GP in the catchment area).

(2) Meet the Diagnostic and Statistical Manual V Criteria for Anorexia Nervosa or OSFED (Other Specified Feeding or Eating Disorder) of Anorexic type.

(3) Are aged 18 or above.

(4) Have BMI >15kg/m^2, are currently stable in weight (i.e. not dropping more than 0.5kg a week), and no severe medical risks (indicated by blood pressure, muscle strength, blood chemistry).

(5) Have sufficient English language abilities to complete a talking therapy.

Service users are excluded if they have/are:

(1) Considerable physical or psychological risk, including active suicidal thoughts and plans.

(2) Comorbidity requiring treatment priority.

(3) Alcohol/substance dependency.

(4) Participating in another treatment trial

(5) Intellectual disabilities

For peer review only - http://bmjopen.bmj.com/site/about/guidelines.xhtml

(6) Pregnant

Due to the nature of a feasibility study, the most severely ill with BMI<15 or at the time of entry into the study will be excluded.

Therapists

Therapists are eligible to offer SPEAKS in the trial if they:

(1) Are a specialist eating disorder therapist > three years experience

(2) Work in Kent All Age Eating Disorder Service or Sussex Eating Disorder Service

(3) Have specialist training in an experiential dialogical self chairwork model (e.g. emotion-focused therapy, schema therapy, compassion-focused therapy)

Interventions

SPEAKS Intervention

SPEAKS is an individual outpatient psychotherapy for adults with AN. Participants receive weekly individual sessions of psychotherapy for 9-12 months with two follow-up sessions within 3 months of completing therapy. SPEAKS is intended to be offered face-to-face in a clinic setting; however, due to the COVID-19 pandemic video sessions via on an online platform will be provided.

SPEAKS is a direct replacement for psychotherapy as usual. Therapists will receive weekly supervision by supervisors trained in the supervision requirements of SPEAKS. All other usual care procedures (e.g. dietician appointments; carer's workshops) will remain, but be reported. All usual service protocols in terms of accessing additional care such as inpatient treatment will remain as per local and national guidance. People will be removed from the trial if they require immediate inpatient treatment at any point or request removal for any reason, and these data will be reported.

Intervention Development. The development of SPEAKS is consistent with MRC guidance for complex interventions[18]. SPEAKS was developed following a clear programme of research to integrate quantitative and qualitative data to achieve an initial model of the presentation (lost

For peer review only - http://bmjopen.bmj.com/site/about/guidelines.xhtml

emotional self)[16] and potential necessary elements for therapeutic change (e.g. Drinkwater et al., in prep). This resulted in a clear hypothesised change process to be targeted in therapy[17]. This diagrammatically represents the intervention, depicting anticipated intervention components, expected mechanisms of change and outcomes. The intervention developers (AO, TL & HS) applied relevant psychological therapy model to target highlighted mechanisms, resulting in an integrative therapy drawing largely from emotion focused therapy (EFT) and schema therapy (ST).

Intervention guidebook. The SPEAKS intervention is written up in a guidebook for therapists to follow. It outlines this change process, mapping it onto expected 'phases' of therapy. In each phase, associated hypothesised mechanisms are outlined and 'therapeutic tasks' described. The guidebook meets requirements for preliminary feasibility evaluation as outlined in the stage model approach for developing a psychotherapy[19].

Therapist training & supervision. In additional to background training in an experiential dialogical self model, therapists receive four days training in the SPEAKS model and therapy. Further regular training days will be organised throughout the trial. Therapists receive ongoing supervision throughout by SPEAKS developers and psychologists working within the clinical services. Where consented to by participants, therapy sessions are video recorded for supervision. Regular reflective practice groups will be offered by a SPEAKS developer and psychologist external to the clinical services (TL).

Treatment fidelity, untoward events and protocol adherence

Reviewing video taped sessions in supervision ensures competent treatment delivery adhering to the SPEAKS model. This is usual good practice in both EFT and ST supervision models. Case records of each therapy session in client notes will outline the phase of therapy, emotions focused upon, and the therapeutic task used.

For peer review only - http://bmjopen.bmj.com/site/about/guidelines.xhtml

For peer review only

Usual service SOPs regarding all clinical care and risk management will be followed. Any protocol violations impacting the delivery of SPEAKS such as admission to hospital will be recorded as per Trust guidance, and also logged in trial records for later reporting. Participants who are admitted to hospital will be withdrawn from the study.

Outcomes

Measures of validity and acceptability. Triangulation of qualitative and quantitative data will address acceptability and validity of SPEAKS to participants and therapists.

Qualitative. Participant and therapist lived experience of SPEAKS, its ability to facilitate targeted change, as well as perceived validity and acceptability, will be examined using post-intervention semi-structured interviews completed by the researcher.

Quantitative. Acceptability and perceived value of core SPEAKS components will be quantitatively assessed using visual analogue scales.

Measures of reach and recruitment**.** Screening of potential participants will be logged and details of unmet inclusion criteria will be anonymously recorded. Conversion rates of people approached agreeing to participate will be monitored. Where participation is declined, reasons will be anonymously recorded. Completeness of measures at each time-point will be reported.

Measures of adherence and compliance. Treatment fidelity strategies will be employed consistent with the treatment fidelity checklist[20], including a clear intervention description (guide-book) and standardised therapist training. As described above, session recordings will enable assessment of adherence to SPEAKS, where consented to by participants. Data regarding length of treatment, number of sessions and session content will be recorded anonymously and assessed.

For peer review only - http://bmjopen.bmj.com/site/about/guidelines.xhtml

Measures for sample size estimation. In line with DSM V criteria for AN and OSFED-AN type (atypical AN) which emphasises rate of weight loss and ED cognitions, the primary outcome measure is of eating disorder cognitions and behaviours; this will be used for sample size estimation. Other clinical and Intervention-specific measures will also be assessed.

Clinical outcomes measures. The primary outcome measure of symptom change for sample size estimation is the Eating Disorder Examination Questionnaire (EDEQ)[21]. Other indications of symptoms, such as BMI (kg/m^2); Depression, Anxiety and Stress Scales 21 (DASS-21)[22] and Clinical Impairment Assessment (CIA)[23] are also collected.

Intervention-specific measures. Beliefs About Emotions Questionnaire[24], Young's Schema Questionnaire–Short form[25], Schema Mode Inventory ED–Short form (SMI-ED-SF)[26]; Silencing the Self Scale[27], The Sense of Agency Scale[28]; and Difficulties with Emotion Regulation Scale (DERS)[29].

Measures of economic evaluation. An adapted version of the Client Socio-demographic and Services Receipt Inventory (CSSRI)[30] will collect economic data pre- and post-intervention to assess treatment costs received in the six months preceding and during SPEAKS.

Measures of SPEAKS Change Process. The following analyses will test the SPEAKS hypothesised therapeutic change process.

(1) Videoed therapy sessions will be analysed according to two coding systems to better understand what change is associated with better clinical outcomes, including hypotheses about both the content and order of change. The Innovative Moments Coding System[31] is a systematic reliable method for identifying innovative moments in therapy, categorising by type (action, reflection, protest, reconceptualization, performing change). The Classification of Affective Meaning States (CAMS)[32] codes the presence of emotions expressed during

For peer review only - http://bmjopen.bmj.com/site/about/guidelines.xhtml

therapy. The CAMS includes 9 categories of emotion: global distress, rejecting anger, fear/shame, negative self-evaluation, unmet need, relief, assertive anger/self-compassion, hurt/grief, and acceptance/agency.

(2) We will apply thematic analysis to psychological formulations of 'schema modes' constructed by participants during therapy as compared against those endorsed in a quantitative measure (SMI-ED; see Intervention Specific Measures). This will enable us to improve the SPEAKS intervention based on the most salient schema modes, and mode changes associated with better outcomes.

Participants may continue to participate without agreeing to their formulations or therapy recordings being analysed.

Participant timeline

Participants will be assessed using quantitative variables examining clinical and emotion change collected pre-intervention, at 3, 6 and 9 months into the intervention and post-intervention (12 months; Figure 2). Collecting questionnaire data every three months is standard practice within the Eating Disorder Services. Due to the coronavirus pandemic, some participants experienced a break in their SPEAKS therapy. In order to ensure that all participants are able to access the full 9-12 months of therapy intended, therapy will be extended for those impacted by a maximum of 3 months. Where relevant, this will be discussed and agreed between therapist and participant. In order to capture end of therapy data for those affected, we will include an extra data collection time-point at 15 months just for these participants.

Qualitative acceptability interviews with service users will be completed at the end of each participant's involvement in the study (at 12 or 15 months follow-up). Therapist acceptability interviews will be completed when they have finished working with their final SPEAKS participant.

For peer review only - http://bmjopen.bmj.com/site/about/guidelines.xhtml

Sample size

The feasibility design must balance precision with unethical exposure of participants to the risks being monitored alongside unnecessary expense[33]. Teare and colleagues[34] recommend 35 participants for sufficient feasibility data and precision of mean and variance. A sample size of 36 has two-sided 80% confidence interval with a width equal to 0.411, when the standard deviation is 1.3 (standard deviation for BMI following MANTRA)[6]. Therapy attrition rates for people with AN can reach 40%[9]. These data suggest an approximate revised sample size of up to 60 participants in order to achieve a sample of 36 participants completing therapy.

Recruitment

Patients will be consecutive referrals meeting inclusion/exclusion criteria to the two sites in South East England: NELFT and SPFT. Participants will be identified from waiting lists in the first instance in order of referral. Recruitment will continue until SPEAKS trained therapists no longer have available therapy spaces and will recommence when further spaces come available.

For peer review only

Methods: Data collection, management, and analysis

Data collection methods & retention

Data at all time-points will be organised and collected by a member of the research team. Mutually agreed appointment times for completion of all measures will be agreed. The researcher will maintain engagement of participants throughout the study with regular newsletters and individual correspondence.

Data management & availability

Data entry errors will be checked by double entering 10% of data. Examining the data for impossible values by looking at data ranges will test data quality. No post-treatment data will be released until the database is locked. The datasets generated and/or analysed during the current study during this study will be included in the subsequent results publication. Anonymised participant level data available on request.

Data analysis

The data will be analysed using password protected NHS computers at NELFT. It will be analysed by the research study team, chiefly the research worker and clinical psychology trainees as part of their doctoral theses. Qualitative data analysis will be completed before quantitative analysis to avoid bias in interpretation.

Methods: Monitoring

Data Monitoring

A Research Steering Committee (RSG) comprised of the research team including CI and PIs, representatives from the sponsors, representatives from both Eating Disorder Services and PPI members (with history of anorexia and family members of those with experience of anorexia) oversees the SPEAKS research programme. It is chaired by an experienced researcher independent from the trial Trusts and sponsor. The RSG meets every six months for reporting and discussion.

For peer review only - http://bmjopen.bmj.com/site/about/guidelines.xhtml

Additional meetings can be convened at short notice at additional times if required. The RSG chair is also available for independent advice to the research team.

Patient and Public Involvement

PPI has been in place since the inception of the study before trial funding was sought. An initial Research Design Service Patient and Public Involvement Grant covered costs of a consultation series to assess SPEAKS model and trial validity. This informed intervention development, study design, and the SPEAKS name. Following trial funding, feedback on key documents such as the plain English summary and participant information sheets (PIS) were obtained via contacts made in this initial consultation. Further, PPI input continues throughout the study via the RSG and in open feedback events at key stages of the study advertised via local charity and support group networks.

Adverse event reporting and harms

Protocol violations such as admission to hospital are recorded as per Trust guidance, and also logged in trial records for later reporting. Participants admitted to hospital will be withdrawn from the study. All research staff will be NIHR Good Clinical Practice (GCP) trained and follow safety reporting procedures in line with these guidelines. Therapists monitor participant safety with regular risk assessments and will report to the PI if they become aware of potential harm to the participant or others. Any events that lead to participants experiencing potential or actual serious harm are recorded by the researcher and reported to the PI and sponsor within 24 hours of knowledge of the event. Decision on the expectedness and relatedness to the study intervention will be taken, with further investigation if appropriate. The PI and sponsor will monitor events in case a pattern emerges, taking action if necessary. Clinical risk will be managed within the ED service guidelines.

Auditing

For peer review only - http://bmjopen.bmj.com/site/about/guidelines.xhtml

For peer review only

Overall study conduct, and study conduct at individual sites, will be monitored by the Sponsor Monitor at regular intervals.

The Trial Master File (TMF) will be audited at site initiation, annually for the duration of the study and at study closure. This audit will follow the sponsor Standard Operating Procedures (SOP) and includes checks of: completeness and secure storage of TMF, study-wide approvals, study-wide safety and deviation/violation reporting, Good Clinical Practice (GCP) compliance and performance of study against recruitment targets.

Individual research sites will be monitored after five participants have been recruited as part of site initiation, annually from site initiation date and at study closure at site. Monitoring will follow the sponsor SOP and include monitoring of the Investigator Site File (ISF) for completeness and secure storage, SDV checks, CRF checks, Serious Adverse Events and deviation/violation checks, GCP compliance and performance of site against recruitment targets.

Ethics and dissemination

Research ethics approval

This study has been reviewed in accordance with the guidelines for Canterbury Christ Church University research and has been approved by the London – Bromley Research Ethics Committee (NHS Rec Reference REC Ref: 19/LO/1530).

Protocol amendments

Any protocol amendments will be communicated to all relevant parties and research sites. Notice of intention to submit an amendment will be provided via email to the sponsor and the research governance representatives at all sites to offer an opportunity to discuss any queries prior to submission and confirm support. Ethical approval will subsequently be sought via online submission following IRAS guidance. Once ethical approval has been granted for an amendment this will be disseminated to all parties and official registries (such as ISRCTN) will be notified.

For peer review only - http://bmjopen.bmj.com/site/about/guidelines.xhtml

Consent

Patients meeting inclusion criteria will be provided with a PIS by their assessing clinician if they are willing to hear more about the study. Informed consent will be obtained at a face to face meeting with a member of the research team. The meeting will take place at least 48 hours after the participant has been provided with the PIS, with additional time given as necessary.

At the consent appointment, the research worker will answer any initial questions from the potential participant. They will review the PIS, highlighting key aspects and checking the patient's understanding of what study involvement entails. The researcher will explicitly state and make clear that deciding not to participate will not affect the patient's care in any way, and that if they decide to take part they can change their mind at any time without affecting their current or future care. The research team (like all other clinical staff members) will be trained in the principles of mental capacity and will hold this in mind throughout. Obtaining consent will be a focus of research supervision.

Confidentiality

In line with usual clinical practice, all participant information will be kept confidential, except as governed by law (i.e. if there is a legal obligation on the researcher to disclose this information to authorities due to risk concerns).

Signed informed consent forms will be stored separately from completed questionnaires and interview transcripts, which will be notated with only a participant number and/or pseudonym. Signed informed consent forms will be kept in a locked cabinet on NELFT or SPFT Property as relevant. Completed questionnaire data will be entered into study databases in encrypted Trust network folders accessible only to the research team. Any video recordings of therapy sessions and formulation descriptions will be stored on secure encrypted password protected NHS hard drives.

For peer review only

For peer review only - http://bmjopen.bmj.com/site/about/guidelines.xhtml

Participant identity will not be included in written interview transcripts, and will not be revealed in any publication resulting from this study. Data gathered from this study will be retained as required by regulations, which is up to ten years following the publication of empirical articles or communications describing the results of the study.

Availability of data and materials

The study databases and TMFs will only be available to the research team employed by the participating Trusts. The trainee psychologists analysing video recordings and psychological formulation data will not know participant identity beyond what they see in recorded therapy sessions. Qualitative interview recordings may be transcribed by a third party agency approved by the sponsor. Participant names and personal details will not be disclosed and full confidentiality agreements will be in place in keeping with all protocols set out herein.

Dissemination policy

Data will be disseminated via high-impact, peer-reviewed journals, with Open Access sought where possible. Papers will be submitted for conference presentations to achieve dissemination to practitioners and professionals within the field of eating disorders. Dissemination to service users and clinical networks is considered extremely important and will be achieved via service user networks across the NHS Trusts and HEI involved in the study, via charity networks (e.g. BEAT) and through a free open conference hosted by the NHS Trusts and HEI to disseminate findings and facilitate feedback on the SPEAKS clinical model and intervention.

For peer review only - http://bmjopen.bmj.com/site/about/guidelines.xhtml

Discussion

This SPEAKS feasibility trial aims to assess validity and acceptability, research and recruitment, sample size estimation and initial economic indications of the SPEAKS intervention for adults with anorexia. It also aims to test the hypothesised change process of the SPEAKS intervention.

Potential implications

This results of this first test of the SPEAKS intervention for adults with AN or Otherwise Specified Feeding or Eating Disorder- AN type will provide initial indication of the feasibility of SPEAKS and inform whether progression to larger trials is appropriate. Change process analysis will provide valuable insights into the hypotheses of SPEAKS, affording important data for the refinement of the intervention, whilst also contributing to the broader evidence-base of relevant change processes for adults with AN.

Strengths

This feasibility trial is strengthened by its multi-site design. Furthermore, sites included are relatively research naïve in the delivery of clinical trials facilitating a realistic insight into the feasibility of SPEAKS intervention delivery within naturalistic NHS services. Inclusion of consecutive referrals enables a broad spectrum of participants across AN and OSFED severity and presentations, increasing generalisability. Robust measures to facilitate adherence to the model are included.

Challenges

SPEAKS is an experiential relational model designed to be delivered in face-to-face settings. This trial began in the wake of the COVID-19 pandemic resulting in therapy sessions moving indefinitely to online video platforms with most participants never meeting their therapist in person. People with AN are often regarded as difficult to engage and have high levels of therapy dropout, thus issues of

For peer review only - http://bmjopen.bmj.com/site/about/guidelines.xhtml

engagement with clinical and research protocols are a challenge even without this context. These factors may affect outcomes or length of delivery.

For peer review only

Declaration of competing interests

Not applicable. The authors confirm there are no interests to declare.

Funding

This feasibility study is funded as part of an Integrated Clinical Academic Fellowship-Clinical Lectureship awarded to AO (ICA-CL-2015-01-005) supported by the National Institute for Health Research and Health Education England. The views expressed in this publication are those of the authors and not necessarily those of the NHS, the National Institute for Health Research, Health Education England or the Department of Health.

Roles and Responsibilities

Author affiliation & Contributions

AO is affiliated with Kent and Medway All Age Eating Disorder Service, North East London NHS Foundation Trust (NELFT), The Courtyard, Pudding Lane, Maidstone, ME14 1PA, United Kingdom and The Salomons Institute for Applied Psychology, Canterbury Christ Church University, Canterbury, United Kingdom. Email: annaoldershaw@hotmail.com Tel: 0300 300 1980

AO is the Chief Investigator and Corresponding Author. She led protocol development and wrote all trial documents. She is co-developer of the SPEAKS intervention, and wrote the SPEAKS intervention guidebook. She is Principal Investigator (PI) for the Kent site.

TL is affiliated with The Salomons Institute for Applied Psychology, Canterbury Christ Church University, Canterbury, United Kingdom. Tony.Lavender@canterbury.ac.uk

TL is co-developer of the SPEAKS intervention and offered feedback and edits on the SPEAKS intervention guidebook. He contributed to the study design and development of the protocol. He is chair of the RSG.

For peer review only - http://bmjopen.bmj.com/site/about/guidelines.xhtml

RB is affiliated with Kent and Medway All Age Eating Disorder Service NELFT.

Randeep.Basra@nelft.nhs.uk

RB is research assistant on the SPEAKS feasibility trial. He has provided input into the protocol and assisted in the preparation and content of this manuscript.

HS is affiliated with Sussex Eating Disorders Service and Research and Development Department, Sussex Partnership NHS Foundation Trust (SPFT), Sussex, United Kingdom.

Helen.Startup@sussexpartnership.nhs.uk HS is co-developer of the SPEAKS intervention and offered feedback and edits on the SPEAKS intervention guidebook. She contributed to the study design and development of the protocol. She is PI for the Sussex site.

All authors read and approved the final manuscript.

For peer review only

References

1. PricewaterhouseCoopers. The costs of eating disorders: Social, health and economic impacts. 2015. https://www.b-eat.co.uk/assets/000/000/302/The_costs_of_eating_disorders_Final_original.pdf Accessed 10 Dec, 2020.
2. Viana MC, Gruber MJ, Shahly V, Alhamzawi A, Alonso J, Andrade LH, Angermeyer MC, Benjet C, Bruffaerts R, Caldas-de-Almeida JM, Girolamo GD. Family burden related to mental and physical disorders in the world: results from the WHO World Mental Health (WMH) surveys. Brazilian Journal of Psychiatry. 2013 Jun;35(2):115-25.
3. National Institute of Health and Care Excellence Guideline. Eating disorders: recognition and treatment. NG69. London: National Institute of Health and Care Excellence. 2017.
4. Carter FA, Jordan J, McIntosh VV, Luty SE, McKenzie JM, Frampton CM, Bulik CM, Joyce PR. The long-term efficacy of three psychotherapies for anorexia nervosa: A randomized, controlled trial. International Journal of eating disorders. 2011 Nov;44(7):647-54.
5. Fairburn CG, Bailey-Straebler S, Basden S, Doll HA, Jones R, Murphy R, O'Connor ME, Cooper Z. A transdiagnostic comparison of enhanced cognitive behaviour therapy (CBT-E) and interpersonal psychotherapy in the treatment of eating disorders. Behaviour research and therapy. 2015 Jul 1;70:64-71.
6. Schmidt, U., Oldershaw, A., Jichi, F., Sternheim, L., Startup, H., McIntosh, V., ... & Landau, S. (2012). Out-patient psychological therapies for adults with anorexia nervosa: randomised controlled trial. *The British Journal of Psychiatry*, *201*(5), 392-399.
7. Schmidt U, Magill N, Renwick B, Keyes A, Kenyon M, Dejong H, Lose A, Broadbent H, Loomes R, Yasin H, Watson C. The Maudsley Outpatient Study of Treatments for Anorexia Nervosa and Related Conditions (MOSAIC): Comparison of the Maudsley Model of Anorexia Nervosa Treatment for Adults (MANTRA) with specialist supportive clinical management (SSCM) in

For peer review only

outpatients with broadly defined anorexia nervosa: A randomized controlled trial. Journal of consulting and clinical psychology. 2015 Aug;83(4):796.

8. Zipfel S, Wild B, Groß G, Friederich HC, Teufel M, Schellberg D, Giel KE, de Zwaan M, Dinkel A, Herpertz S, Burgmer M. Focal psychodynamic therapy, cognitive behaviour therapy, and optimised treatment as usual in outpatients with anorexia nervosa (ANTOP study): randomised controlled trial. The Lancet. 2014 Jan 11;383(9912):127-37.
9. DeJong H, Broadbent H, Schmidt U. A systematic review of dropout from treatment in outpatients with anorexia nervosa. International Journal of Eating Disorders. 2012 Jul;45(5):635-47.
10. Bulik CM. The challenges of treating anorexia nervosa. Lancet (London, England). 2014 Jan 11;383(9912):105.
11. Startup H, Mountford V, Lavender A, Schmidt U. Cognitive behavioural case formulation in complex eating disorders. Case formulation in cognitive behaviour therapy: The treatment of challenging and complex cases. 2015 Jul 6:239-64.
12. Kass AE, Kolko RP, Wilfley DE. Psychological treatments for eating disorders. Current opinion in psychiatry. 2013 Nov;26(6):549.
13. Sala M, Heard A, Black EA. Emotion-focused treatments for anorexia nervosa: a systematic review of the literature. Eating and Weight Disorders-Studies on Anorexia, Bulimia and Obesity. 2016 Jun 1;21(2):147-64.
14. Oldershaw A, Lavender T, Schmidt U. Are socio-emotional and neurocognitive functioning predictors of therapeutic outcomes for adults with anorexia nervosa?. European Eating Disorders Review. 2018 Jul;26(4):346-59.
15. Kendler KS, Campbell J. Interventionist causal models in psychiatry: repositioning the mind-body problem. Psychological medicine. 2009 Jun 1;39(6):881.

For peer review only

For peer review only - http://bmjopen.bmj.com/site/about/guidelines.xhtml

16. Oldershaw A, Startup H, Lavender T. Anorexia Nervosa and a lost emotional self: A psychological formulation of the development, maintenance, and treatment of Anorexia Nervosa. Frontiers in Psychology. 2019 Mar 4;10:219.
17. Oldershaw A, Startup H. 18 Building the Healthy Adult in eating disorders. Creative Methods in Schema Therapy: Advances and Innovation in Clinical Practice. 2020 Jun 18.
18. Craig P, Dieppe P, Macintyre S, Michie S, Nazareth I, Petticrew M. Developing and evaluating complex interventions: the new Medical Research Council guidance. Bmj. 2008 Sep 29;337.
19. Carroll KM, Nuro KF. One size cannot fit all: A stage model for psychotherapy manual development. Clinical Psychology: Science and Practice. 2002 Dec;9(4):396-406.
20. Borelli B, Sepinwall D, Ernst D, Bellg AJ, Czajkowski S, Breger R, DeFrancesco C, Levesque C, Sharp DL, Ogedegbe G, Resnick B. A new tool to assess treatment fidelity and evaluation of treatment fidelity across 10 years of health behavior research. Journal of consulting and clinical psychology. 2005 Oct;73(5):852.
21. Fairburn CG, Beglin SJ. Eating disorder examination questionnaire. Cognitive behavior therapy and eating disorders. 2008 Sep:309-13.
22. Henry JD, Crawford JR. The short-form version of the Depression Anxiety Stress Scales (DASS-21): Construct validity and normative data in a large non-clinical sample. British journal of clinical psychology. 2005 Jun;44(2):227-39.
23. Bohn K, Fairburn CG. The clinical impairment assessment questionnaire (CIA). Cognitive behavioral therapy for eating disorders. 2008:1-3.
24. Manser R, Cooper M, Trefusis J. Beliefs about emotions as a metacognitive construct: Initial development of a self-report questionnaire measure and preliminary investigation in relation to emotion regulation. Clinical Psychology & Psychotherapy. 2012 May;19(3):235-46.
25. Young JE, Brown G. Young Schema Questionnaire-Short Form; Version 3. Psychological Assessment. 2005.

For peer review only

26. Simpson SG, Pietrabissa G, Rossi A, Seychell T, Manzoni GM, Munro C, Nesci JB, Castelnuovo G. Factorial structure and preliminary validation of the Schema Mode Inventory for Eating Disorders (SMI-Ed). Frontiers in psychology. 2018 Apr 24;9:600.
27. Jack DC. The silencing the self scale. Fairhaven Faculty Publications. 2017.
28. Tapal A, Oren E, Dar R, Eitam B. The sense of agency scale: A measure of consciously perceived control over one's mind, body, and the immediate environment. Frontiers in psychology. 2017 Sep 12;8:1552.
29. Gratz KL, Roemer L. Multidimensional assessment of emotion regulation and dysregulation: Development, factor structure, and initial validation of the difficulties in emotion regulation scale. Journal of psychopathology and behavioral assessment. 2004 Mar 1;26(1):41-54.
30. Chisholm D, Knapp MR, Knudsen HC, Amaddeo F, Gaite LU, Van Wijngaarden BO, EPSILON Study Group. Client socio-demographic and service receipt inventory–European version: development of an instrument for international research: EPSILON Study 5. The British Journal of Psychiatry. 2000 Jul;177(S39):s28-33.
31. Gonçalves MM, Ribeiro AP, Mendes I, Matos M, Santos A. Tracking novelties in psychotherapy process research: The innovative moments coding system. Psychotherapy research. 2011 Sep 1;21(5):497-509.
32. Pascual-Leone A, Greenberg LS. Classification of affective-meaning states. A. Pascual-Leone, Emotional processing in the therapeutic hour: Why "the only way out is through. 2005:289-367.
33. Billingham SA, Whitehead AL, Julious SA. An audit of sample sizes for pilot and feasibility trials being undertaken in the United Kingdom registered in the United Kingdom Clinical Research Network database. BMC medical research methodology. 2013 Dec;13(1):1-6.
34. Teare MD, Dimairo M, Shephard N, Hayman A, Whitehead A, Walters SJ. Sample size requirements to estimate key design parameters from external pilot randomised controlled trials: a simulation study. Trials. 2014 Dec;15(1):1-3.

For peer review only - http://bmjopen.bmj.com/site/about/guidelines.xhtml

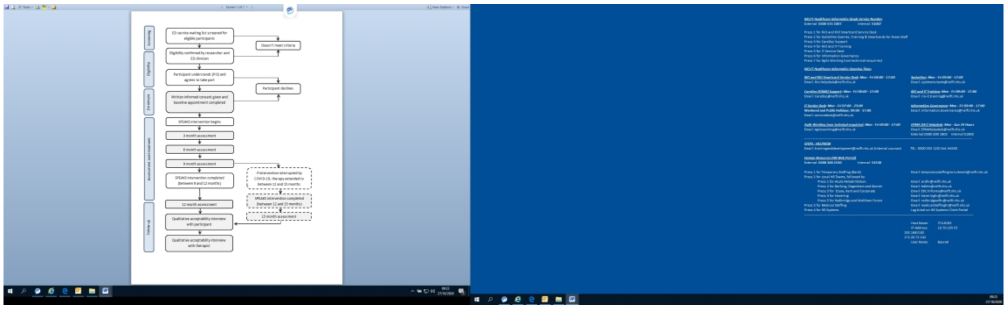

Figure 1. Consort diagram of SPEAKS feasibility trial design.

1270x381mm (72 x 72 DPI)

		SPEAKS STUDY PERIOD				
	Enrolment	Post-enrolment				Post-intervention
TIMEPOINT	$-t_1$	Baseline	3 months	6 months	9 months	12-15 months
ENROLMENT:						
Eligibility screen	X					
Informed consent	X					
INTERVENTION:						
SPEAKS ASSESSMENTS						
Adapted CSSRI		X				X
Clinical outcome measures		X	X	X	X	X
Intervention-specific measures		X	X	X	X	X
Qualitative interviews						X

Figure 2: SPIRIT schedule of enrolment, intervention, and assessments for the SPEAKS feasibility trial.

SPIRIT
STANDARD PROTOCOL ITEMS: RECOMMENDATIONS FOR INTERVENTIONAL TRIALS

SPIRIT 2013 Checklist: Recommended items to address in a clinical trial protocol and related documents

Items in bold and indicated with * are included in the SPEAKS study protocol paper submission. Any items which are not included were not deemed relevant to the SPEAKS study design.

For peer review only

**Section/item**	**Item No**	**Description**
**Administrative information**		
***Title**	**1**	Descriptive title identifying the study design, population, interventions, and, if applicable, trial acronym
***Trial registration**	**2a**	Trial identifier and registry name. If not yet registered, name of intended registry
	2b	All items from the World Health Organization Trial Registration Data Set
***Protocol version**	**3**	Date and version identifier
***Funding**	**4**	Sources and types of financial, material, and other support
***Roles and responsibilities**	**5a**	Names, affiliations, and roles of protocol contributors
	**5b**	Name and contact information for the trial sponsor
	**5c**	Role of study sponsor and funders, if any, in study design; collection, management, analysis, and interpretation of data; writing of the report; and the decision to submit the report for publication, including whether they will have ultimate authority over any of these activities

For peer review only - http://bmjopen.bmj.com/site/about/guidelines.xhtml

	**5d**	Composition, roles, and responsibilities of the coordinating centre, steering committee, endpoint adjudication committee, data management team, and other individuals or groups overseeing the trial, if applicable (see Item 21a for data monitoring committee)

Introduction

***Background and rationale**	**6a**	Description of research question and justification for undertaking the trial, including summary of relevant studies (published and unpublished) examining benefits and harms for each intervention
	6b	Explanation for choice of comparators
***Objectives**	**7**	Specific objectives or hypotheses
***Trial design**	**8**	Description of trial design including type of trial (eg, parallel group, crossover, factorial, single group), allocation ratio, and framework (eg, superiority, equivalence, noninferiority, exploratory)

Methods: Participants, interventions, and outcomes

***Study setting**	**9**	Description of study settings (eg, community clinic, academic hospital) and list of countries where data will be collected. Reference to where list of study sites can be obtained
***Eligibility criteria**	**10**	Inclusion and exclusion criteria for participants. If applicable, eligibility criteria for study centres and individuals who will perform the interventions (eg, surgeons, psychotherapists)
***Interventions**	**11a**	Interventions for each group with sufficient detail to allow replication, including how and when they will be administered
	**11b**	Criteria for discontinuing or modifying allocated interventions for a given trial participant (eg, drug dose change in response to harms, participant request, or improving/worsening disease)
	**11c**	Strategies to improve adherence to intervention protocols, and any procedures for monitoring adherence (eg, drug tablet return, laboratory tests)
	**11d**	Relevant concomitant care and interventions that are permitted or prohibited during the trial

For peer review only - http://bmjopen.bmj.com/site/about/guidelines.xhtml

***Outcomes**	**12**	Primary, secondary, and other outcomes, including the specific measurement variable (eg, systolic blood pressure), analysis metric (eg, change from baseline, final value, time to event), method of aggregation (eg, median, proportion), and time point for each outcome. Explanation of the clinical relevance of chosen efficacy and harm outcomes is strongly recommended
***Participant timeline**	**13**	Time schedule of enrolment, interventions (including any run-ins and washouts), assessments, and visits for participants. A schematic diagram is highly recommended (see Figure)
***Sample size**	**14**	Estimated number of participants needed to achieve study objectives and how it was determined, including clinical and statistical assumptions supporting any sample size calculations
***Recruitment**	**15**	Strategies for achieving adequate participant enrolment to reach target sample size

Methods: Assignment of interventions (for controlled trials)

Allocation:

Sequence generation	16a	Method of generating the allocation sequence (eg, computer-generated random numbers), and list of any factors for stratification. To reduce predictability of a random sequence, details of any planned restriction (eg, blocking) should be provided in a separate document that is unavailable to those who enrol participants or assign interventions
Allocation concealment mechanism	16b	Mechanism of implementing the allocation sequence (eg, central telephone; sequentially numbered, opaque, sealed envelopes), describing any steps to conceal the sequence until interventions are assigned
Implementation	16c	Who will generate the allocation sequence, who will enrol participants, and who will assign participants to interventions
Blinding (masking)	17a	Who will be blinded after assignment to interventions (eg, trial participants, care providers, outcome assessors, data analysts), and how
	17b	If blinded, circumstances under which unblinding is permissible, and procedure for revealing a participant's allocated intervention during the trial

Methods: Data collection, management, and analysis

***Data collection methods**	**18a**	Plans for assessment and collection of outcome, baseline, and other trial data, including any related processes to promote data quality (eg, duplicate measurements, training of assessors) and a description of study instruments (eg, questionnaires, laboratory tests) along with their reliability and validity, if known. Reference to where data collection forms can be found, if not in the protocol
	18b	Plans to promote participant retention and complete follow-up, including list of any outcome data to be collected for participants who discontinue or deviate from intervention protocols
***Data management**	**19**	Plans for data entry, coding, security, and storage, including any related processes to promote data quality (eg, double data entry; range checks for data values). Reference to where details of data management procedures can be found, if not in the protocol
***Statistical methods**	**20a**	Statistical methods for analysing primary and secondary outcomes. Reference to where other details of the statistical analysis plan can be found, if not in the protocol
	20b	Methods for any additional analyses (eg, subgroup and adjusted analyses)
	20c	Definition of analysis population relating to protocol non-adherence (eg, as randomised analysis), and any statistical methods to handle missing data (eg, multiple imputation)

Methods: Monitoring

***Data monitoring**	**21a**	Composition of data monitoring committee (DMC); summary of its role and reporting structure; statement of whether it is independent from the sponsor and competing interests; and reference to where further details about its charter can be found, if not in the protocol. Alternatively, an explanation of why a DMC is not needed
	21b	Description of any interim analyses and stopping guidelines, including who will have access to these interim results and make the final decision to terminate the trial
***Harms**	**22**	Plans for collecting, assessing, reporting, and managing solicited and spontaneously reported adverse events and other unintended effects of trial interventions or trial conduct

For peer review only - http://bmjopen.bmj.com/site/about/guidelines.xhtml

***Auditing**	**23**	Frequency and procedures for auditing trial conduct, if any, and whether the process will be independent from investigators and the sponsor

Ethics and dissemination

***Research ethics approval**	**24**	Plans for seeking research ethics committee/institutional review board (REC/IRB) approval
***Protocol amendments**	**25**	Plans for communicating important protocol modifications (eg, changes to eligibility criteria, outcomes, analyses) to relevant parties (eg, investigators, REC/IRBs, trial participants, trial registries, journals, regulators)
***Consent or assent**	**26a**	Who will obtain informed consent or assent from potential trial participants or authorised surrogates, and how (see Item 32)
	26b	Additional consent provisions for collection and use of participant data and biological specimens in ancillary studies, if applicable
***Confidentiality**	**27**	How personal information about potential and enrolled participants will be collected, shared, and maintained in order to protect confidentiality before, during, and after the trial
***Declaration of interests**	**28**	Financial and other competing interests for principal investigators for the overall trial and each study site
***Access to data**	**29**	Statement of who will have access to the final trial dataset, and disclosure of contractual agreements that limit such access for investigators
Ancillary and post-trial care	30	Provisions, if any, for ancillary and post-trial care, and for compensation to those who suffer harm from trial participation
***Dissemination policy**	**31a**	Plans for investigators and sponsor to communicate trial results to participants, healthcare professionals, the public, and other relevant groups (eg, via publication, reporting in results databases, or other data sharing arrangements), including any publication restrictions
	**31b**	Authorship eligibility guidelines and any intended use of professional writers
	**31c**	Plans, if any, for granting public access to the full protocol, participant-level dataset, and statistical code

For peer review

For peer review only - http://bmjopen.bmj.com/site/about/guidelines.xhtml

Appendices

Informed consent materials	32	Model consent form and other related documentation given to participants and authorised surrogates
Biological specimens	33	Plans for collection, laboratory evaluation, and storage of biological specimens for genetic or molecular analysis in the current trial and for future use in ancillary studies, if applicable

*It is strongly recommended that this checklist be read in conjunction with the SPIRIT 2013 Explanation & Elaboration for important clarification on the items. Amendments to the protocol should be tracked and dated. The SPIRIT checklist is copyrighted by the SPIRIT Group under the Creative Commons "Attribution-NonCommercial-NoDerivs 3.0 Unported" license.

For peer review only

BMJ Open

The SPEAKS study: Study protocol of a multisite feasibility trial of the Specialist Psychotherapy with Emotion for Anorexia in Kent and Sussex (SPEAKS) intervention for outpatients with anorexia nervosa or otherwise specified feeding and eating disorders, anorexia nervosa type

Journal:	*BMJ Open*
Manuscript ID	bmjopen-2021-050350.R1
Article Type:	Protocol
Date Submitted by the Author:	23-Sep-2021
Complete List of Authors:	Oldershaw, Anna; North East London NHS Foundation Trust Goodmayes Hospital, Kent and Medway All Age Eating Disorder Service; Canterbury Christ Church University Faculty of Social and Applied Sciences, Salomons Institute for Applied Psychology<br>Lavender, Tony; Canterbury Christ Church University, Salomons Institute for Applied Psychology<br>Basra, Randeep; North East London NHS Foundation Trust Goodmayes Hospital, Kent and Medway All Age Eating Disorder Service<br>Startup, Helen; Sussex Partnership NHS Foundation Trust, Brighton and Hove Eating Disorder Service
**Primary Subject Heading**:	Mental health
Secondary Subject Heading:	Evidence based practice, Research methods
Keywords:	Eating disorders < PSYCHIATRY, Adult psychiatry < PSYCHIATRY, QUALITATIVE RESEARCH

SCHOLARONE™
Manuscripts

The SPEAKS study: Study protocol of a multisite feasibility trial of the Specialist Psychotherapy with Emotion for Anorexia in Kent and Sussex (SPEAKS) intervention for outpatients with anorexia nervosa or otherwise specified feeding and eating disorders, anorexia nervosa type

Anna Oldershaw

Kent and Medway All Age Eating Disorder Service, North East London NHS Foundation Trust (NELFT), The Courtyard, Pudding Lane, Maidstone, ME14 1PA, United Kingdom and The Salomons Institute for Applied Psychology, Canterbury Christ Church University, Canterbury, United Kingdom.

Email: anna.oldershaw@nelft.nhs.uk Tel: 0300 300 1980

Tony Lavender

The Salomons Institute for Applied Psychology, Canterbury Christ Church University, Canterbury, United Kingdom.

Randeep Basra

Kent and Medway All Age Eating Disorder Service North East London NHS Foundation Trust (NELFT), Maidstone, United Kingdom.

Helen Startup

Sussex Eating Disorders Service and Research and Development Department, Sussex Partnership NHS Foundation Trust (SPFT), Sussex, United Kingdom.

Keywords: Anorexia nervosa, eating disorders, emotions, emotion focused therapy, schema therapy

Word Count: 3999

For peer review only - http://bmjopen.bmj.com/site/about/guidelines.xhtml

Abstract

Introduction

Anorexia nervosa (AN) is a severe mental health condition associated with high mortality rates and significantly impaired quality of life. National guidelines outline psychotherapeutic interventions as treatments of choice for adults with AN, but outcomes are limited and therapy drop-out high, resulting in calls for new innovative treatments. The Specialist Psychotherapy with Emotion for Anorexia in Kent and Sussex (SPEAKS) research programme sought to develop the SPEAKS intervention avoiding some difficulties inherent in development of earlier interventions, such unclear hypotheses about change processes. SPEAKS focuses on a core hypothesized maintaining factor (emotional experience) with clear proposed model of change. The current feasibility trial aims to provide an initial test of SPEAKS and inform design of a full randomised controlled trial protocol.

Methods & Analysis

This study employs a multi-site, single-arm, within-group, mixed-methods design. Up to 60 participants (36 therapy completers) meeting inclusion criteria will be offered the SPEAKS intervention instead of treatment-as-usual (TAU). SPEAKS is a weekly psychotherapy lasting nine to 12 months, provided by trained and experienced eating disorders therapists. All other clinical input remains inline with TAU. *Acceptability* will be assessed using VAS scales and end of therapy interview. *Reach and recruitment*, such as recruitment yield, will be monitored. To support *sample size estimation* and *economic estimation*, data pertaining to eating disorder-related symptoms will be recorded every three months, alongside service usage and intervention-specific measures. Videoed therapy sessions will inform model adherence. Additional analyses coding videoed therapy will test SPEAKS change process hypotheses.

Ethics & Dissemination

For peer review only - http://bmjopen.bmj.com/site/about/guidelines.xhtml

Ethical approval has been granted by London–Bromley Research Ethics Committee (NHS Rec Reference: 19/LO/1530). Data will be disseminated via high-impact, peer-reviewed journals (Open Access preferred), conferences, service user and charity networks (e.g. UK charity BEAT) and through a free open conference hosted by NHS Trusts and Higher Education Institutions (HEI).

Article summary: Strengths and Limitations

- This feasibility trial studies a novel psychotherapy for adults with AN, with a focus on emotional change: The SPEAKS intervention.
- This feasibility study is strengthened by its multi-site trial design, completed in largely 'research naïve' NHS services, increasing insight into its feasibility and generalisability.
- The study is limited by its single arm design and non-blinded procedures, which may result in biases such as allegiance effects and does not allow for estimating acceptability of a randomised design.
- In addition to its feasibility aims regarding the SPEAKS intervention, the trial design includes analysis of the therapeutic change process with wider implications for understanding therapeutic change for adults with AN.
- The trial has been subject to the challenges imposed by the COVID-19 pandemic including regarding clinical and research engagement.

For peer review only

For peer review only - http://bmjopen.bmj.com/site/about/guidelines.xhtml

Trial registration

Registered on ISRCTN trials registry reference ISRCTN11778891. Prospectively applied 28th November 2019; retrospectively assigned 20th January 2020.

http://www.isrctn.com/ISRCTN11778891

Trial Status

Recruitment began on 12th December 2019 and ends 28th February 2021. All data will be collected and the trial ended by 28th February 2022.

Protocol version

SPEAKS protocol version 3.0 (30/08/2020). Changed were made to the original protocol due to the COVID-19 pandemic. A further set of changes were made to incorporate the measures of change processes, resulting in this being the third version of the protocol.

Trial sponsor information

North East London NHS Foundation Trust

Research and Development Department

1st Floor, Maggie Lilley Suite

Goodmayes Hospital

Barley Lane

Ilford

IG3 8XJ

+44 (0)300 555 1200

Fiona.Horton@NELFT.nhs.uk

For peer review only

Introduction

Background and rationale

Anorexia nervosa (AN) is a severe mental illness with poor prognosis and the highest mortality rate of any psychiatric disorder [1]. Early intervention is key, but is impeded by a delay of around 18 months from symptoms emerging to treatment, and with multiple relapses likely[2]. Caregiving for somebody with severe AN is almost twice as lengthy as for somebody with a physical health disorder (e.g., cancer) or other mental health difficulty (e.g., psychosis)[3]. Thus significant costs exist financially and emotionally to the individual, family and carers, as well as society as a whole, offering a "compelling case for change" in services and treatment[2, p. 9].

Most recent NICE guidelines in the UK recommend that psychotherapeutic interventions be considered for adults with AN[4]. Yet results from randomized controlled trials (RCTs) indicate that out-patient treatments do not out-perform each other or control comparisons[5-9] with weight-restoration achieved by 20 percent of patients after one year[7-9]. AN is highly valued by sufferers resulting in poor treatment engagement and high dropout rates[10]. Thus there is an urgent need to develop innovative interventions that can engage people with AN[4, 11, 12], while targeting unique factors involved in its development and maintenance[13]. Emotional experience has long been recognised as a factor in the development and maintenance of AN and is recognised as a promising target area for therapies[14] and increasingly incorporated into psychotherapy interventions for adults with AN[5-9]. However, outcomes remain limited and it is unclear to what extent emotional difficulties are targeted by these interventions[15].

This trial comprises part of the SPEAKS programme which aimed to develop and test in a feasibility study an emotion focused intervention for adults with anorexia (the SPEAKS intervention). This research programme sought to overcome several key difficulties with the development and application of some earlier interventions, such as unsuccessful targeting of variables or lack of clarity

For peer review only - http://bmjopen.bmj.com/site/about/guidelines.xhtml

on how change is achieved. The SPEAKS programme proposes focussing on a core clearly defined model with one key putative maintaining process (emotional experience in AN) and following an ‘interventionist-causal model approach’[16]. By developing a new model of the development and maintenance of AN drawn from integration of quantitative and qualitative research, it seeks to ensure an emotions’ focus[17]. The SPEAKS intervention hypothesises a clear and testable change process to be targeted in therapy[18; Figure 1]; thus affording the ability to examine proposed mechanisms of change enabling further evidence-based development and refinement of the model. This body of work involved close partnering with stakeholders including those with current and past experience of AN, families, therapists and service managers.

Objectives

This multi-site feasibility trial aims to investigate the SPEAKS intervention in the following domains:

- Acceptability
- Reach and recruitment
- Adherence and compliance
- Sample size & economic evaluation for a potential future efficacy/effectiveness trial
- Change process analysis

We hypothesise that:

(1) SPEAKS will be acceptable to participants and therapists.

(2) SPEAKS will meet sufficient reach and recruitment expectations to support progression to full effectiveness/efficacy trial.

(3) Therapists and service users will be able to adhere to the therapy model as intended and the research requirements

For peer review only - http://bmjopen.bmj.com/site/about/guidelines.xhtml

(4) Participant change over time will reflect the proposed emotion change process outlined in Figure 1.

Trial design

The SPEAKS study is a multi-site, single-armed, within-group mixed-methods design.

For peer review only

Methods: Participants, interventions and outcomes

Study setting

This feasibility study runs in two outpatient specialist eating disorders services (EDS) within the UK National Health Service (NHS): Kent & Medway All Age Eating Disorder Service at NELFT and Sussex Eating Disorder Service at SPFT.

Eligibility criteria

Service Users

Service users are eligible to participate if they:

(1) Are referred into Kent or Sussex EDS and meet service criteria (e.g. registered with a local GP).

(2) Meet Diagnostic and Statistical Manual 5 Criteria for Anorexia Nervosa or OSFED (Other Specified Feeding or Eating Disorder) of Anorexic type.

(3) Are aged 18 or above.

(4) Have BMI >15kg/m^2

(5) Have sufficient English language abilities to complete a talking therapy.

Service users are excluded if they have/are:

(1) Rated as 'High Risk', or as 'High Concern' in weight criteria, on the MARSIPAN Guidelines for adults with eating disorders (i.e. BMI<15; weight loss>500g for 2 consecutive weeks)[19]

(2) Considerable psychological risk, including active suicidal thoughts and plans.

(3) Comorbidity requiring treatment priority.

(4) Alcohol/substance use disorder.

(5) Participating in another treatment trial

(6) Diagnosed Intellectual disability impeding ability to utilise therapy

For peer review only - http://bmjopen.bmj.com/site/about/guidelines.xhtml

(7) Pregnant

Therapists

Therapists are eligible to offer SPEAKS in the trial if they:

(1) Are a specialist eating disorder therapist > three years experience

(2) Work in Kent All Age Eating Disorder Service or Sussex Eating Disorder Service

(3) Have specialist training in an experiential dialogical self chairwork model (e.g. emotion-focused therapy, schema therapy, compassion-focused therapy)

Interventions

SPEAKS Intervention

SPEAKS is an individual outpatient psychotherapy for adults with AN. Participants receive weekly individual sessions of psychotherapy for 9-12 months with two follow-up sessions within 3 months of completion. SPEAKS is intended to be offered face-to-face in a clinic setting; however, due to the COVID-19 pandemic video sessions via on an online platform are provided.

SPEAKS is a direct replacement for psychotherapy as usual. Therapists receive fortnightly supervision by supervisors trained in the supervision requirements of SPEAKS. The services at both sites are in the same region of the UK and follow national NHS treatment guidelines. All usual care procedures, including additional interventions such as dietician appointments or inpatient referrals will remain the same, but be reported. People will be removed from the trial if they require immediate inpatient treatment at any point or request removal for any reason, and these data will be reported.

Intervention Development. The development of SPEAKS is consistent with MRC guidance for complex interventions[20]. SPEAKS was developed following a clear programme of research to integrate quantitative and qualitative data to achieve an initial model of the presentation (lost emotional self)[17] and potential necessary elements for therapeutic change (e.g. Drinkwater et al., in prep). This resulted in a clear hypothesised change process to be targeted in therapy[17]. SPEAKS

For peer review only - http://bmjopen.bmj.com/site/about/guidelines.xhtml

is organised into five phases, with associated mechanisms of change and therapeutic 'tasks' (see Figure 2 for brief overview). The intervention developers (AO, TL & HS) applied relevant psychological therapy models to target highlighted mechanisms, resulting in an integrative therapy drawing chiefly from emotion focused therapy (EFT) and schema therapy (ST). The therapy thus relies on dialogical self theory, and therapeutic tasks are those well established in EFT and ST, such as 'chairwork' interventions to enable 'parts of self' to communicate.

Intervention guidebook. The SPEAKS intervention is written up in a guidebook for therapists to follow. It outlines SPEAKS change process, mapping it onto expected 'phases' of therapy (Figure 2). In each phase, associated hypothesised mechanisms are outlined and 'therapeutic tasks' described. The guidebook meets requirements for preliminary feasibility evaluation as outlined in the stage model approach for developing a psychotherapy[21] and assists intervention adherence.

Therapist training & supervision. In additional to background training in an experiential dialogical self model, therapists receive four days SPEAKS model and therapy training, with further regular training days organised throughout the trial. Ongoing fortnightly supervision is delivered by SPEAKS developers. Where participants consent, all therapy sessions are video recorded for supervision. Regular reflective practice groups are offered by a SPEAKS developer and psychologist external to the clinical services (TL).

Treatment fidelity, untoward events and protocol adherence

Reviewing videotaped sessions in supervision ensures competent treatment delivery adhering to the SPEAKS model. This is usual good practice in both EFT and ST supervision models. Case records of each therapy session in client notes outline the phase of therapy, emotions focused upon, and therapeutic tasks employed.

For peer review only - http://bmjopen.bmj.com/site/about/guidelines.xhtml

Usual service SOPs regarding clinical care and risk management will be followed. Any protocol violations impacting delivery of SPEAKS such as admission to hospital will be recorded as per Trust guidance, and also logged in trial records for later reporting. Participants who are admitted to hospital will be withdrawn from the study.

Outcomes

Measures of acceptability. Triangulation of qualitative and quantitative data will address acceptability of SPEAKS to participants and therapists.

Qualitative. Participant and therapist lived experience of SPEAKS will be examined using post-intervention semi-structured interviews adapted from the Client Change Interview[22], completed by the researcher. Acceptability of aspects of the current and future trial designs are also addressed including use of questionnaires and willingness to be randomised to treatment arms.

Quantitative. Acceptability and perceived value of core SPEAKS components will be quantitatively assessed using visual analogue scales created for the study using questions based on the acceptability interview, and rated on a seven-point scale strongly agree to strongly disagree (e.g. I think the focus of SPEAKS makes sense for me and my difficulties). Numbers of people who choose to end their therapy because they do not think it is acceptable will also be calculated.

Measures of reach and recruitment**.** Screening of potential participants will be logged, and details of unmet inclusion criteria anonymously recorded. Recruitment yield will be monitored. Where participation is declined, reasons will be anonymously recorded. Completeness of measures at each time-point will be reported.

Measures of adherence and compliance. Treatment fidelity strategies will be employed consistent with the treatment fidelity checklist[23], including a clear intervention description (guide-book) and standardised therapist training. As described, session recordings will enable assessment of

For peer review only - http://bmjopen.bmj.com/site/about/guidelines.xhtml

adherence to SPEAKS, where consented to. A random selection of therapy tapes will be assessed for fidelity and adherence to the model judged by the intervention developers. They will assess core components of the treatments such as empathy (using the Therapist Empathy Scale [24]), appropriate task selection based on identified tasks markers, and appropriate task resolution. Data regarding treatment length, session numbers and content will be reported.

Measures for sample size estimation.

Clinical outcomes measures. In line with DSM V criteria for AN and OSFED-AN type (atypical AN) which emphasises rate of weight loss and ED cognitions, the primary outcome measure is of eating disorder cognitions and behaviours for sample size estimation - Eating Disorder Examination Questionnaire (EDEQ)[25]. Other indications of symptoms, such as BMI (kg/m^2); Depression, Anxiety and Stress Scales 21 (DASS-21)[26] and Clinical Impairment Assessment (CIA)[27] are also collected.

Intervention-specific measures. Other clinical and Intervention-specific measures will also be assessed as follows: Beliefs About Emotions Questionnaire[28], Young's Schema Questionnaire–Short form[29], Schema Mode Inventory ED–Short form (SMI-ED-SF)[30]; Silencing the Self Scale[31], The Sense of Agency Scale[32]; and Difficulties with Emotion Regulation Scale (DERS)[33].

Measures of economic evaluation. An adapted version of the Client Socio-demographic and Services Receipt Inventory (CSSRI)[34] will collect economic data pre- and post-intervention to assess treatment costs received in the six months preceding and during SPEAKS.

Measures of SPEAKS Change Process. The following analyses will test hypothesised therapeutic change process.

(1) Videoed therapy sessions will be analysed according to two coding systems to better understand change associated with better clinical outcomes, including hypotheses about both content and order of change. The Innovative Moments Coding System[35] is a

For peer review only - http://bmjopen.bmj.com/site/about/guidelines.xhtml

For peer review only

systematic reliable method for identifying innovative moments (IMs) in therapy, categorising by type (action, reflection, protest, reconceptualization, performing change). IMs are measured by the percentage of time spent elaborating on each IM (temporal salience). The Classification of Affective Meaning States (CAMS)[36] codes the presence of emotions expressed during therapy videos in one minute segments. The CAMS includes nine emotion categories: global distress (rejecting anger, fear/shame, negative self-evaluation, unmet need, relief, assertive anger/self-compassion, hurt/grief, and acceptance/agency) which will be analysed for temporal patterns in the expression of emotion codes over time.

(2) Thematic analysis will be applied to psychological formulations of 'schema modes' constructed by participants during therapy as compared against those endorsed in a quantitative measure (SMI-ED[30]). This will enable us to improve the SPEAKS intervention based on the most salient schema modes, and mode changes associated with better outcomes.

Participants may participate without agreeing to their formulations or therapy recordings being analysed.

Participant timeline

Participants will be assessed using quantitative variables examining clinical and emotion change collected pre-intervention, at 3, 6 and 9 months into the intervention and post-intervention (12 months; Figure 3 and 4). Collecting questionnaire data every three months is standard practice within the Eating Disorder Services. Due to the coronavirus pandemic, some participants experienced a break in their SPEAKS therapy. In order to ensure that all participants are able to access the full 9-12 months of therapy intended, therapy will be extended for those impacted by a maximum of 3 months. Where relevant, this will be discussed and agreed between therapist and participant. In order to capture end of therapy data for those affected, we will include an extra data collection time-point at 15 months just for these participants.

For peer review only - http://bmjopen.bmj.com/site/about/guidelines.xhtml

Qualitative acceptability interviews with service users will be completed at the end of each participant's involvement in the study (at 12 or 15 months follow-up). Therapist acceptability interviews will be completed when they have finished working with their final SPEAKS participant.

Sample size

The feasibility design must balance precision with unethical exposure of participants to the risks being monitored alongside unnecessary expense[37]. Teare and colleagues[38] recommend 35 participants for sufficient feasibility data and precision of mean and variance. A sample size of 36 has two-sided 80% confidence interval with a width equal to 0.411, when the standard deviation is 1.3 (standard deviation for BMI following MANTRA)[7]. Therapy attrition rates for people with AN can reach 40%[9]. These data suggest an approximate revised sample size of up to 60 participants in order to achieve a sample of 36 participants completing therapy.

Recruitment

Patients will be consecutive referrals meeting inclusion/exclusion criteria to the two sites in South-East England: NELFT and SPFT. Participants will be identified from waiting lists in the first instance in order of referral. Recruitment will continue until SPEAKS therapists no longer have available spaces and will recommence when further spaces come available. Recruitment ended in February 2021. This was after journal submission, but prior to completion of peer reviews.

For peer review only - http://bmjopen.bmj.com/site/about/guidelines.xhtml

Methods: Data collection, management, and analysis

Allocation, allocation concealment and blinding

Not applicable due to the single arm design.

Data collection methods & retention

Informed consent and data at all time-points will be organised and collected by a research assistant specifically employed to complete this role. Mutually agreed appointment times for completion of all measures will be agreed. The research assistant will maintain engagement of participants with regular newsletters and individual correspondence.

Data management & availability

Data entry errors will be checked by double entering 10% of data. Examining data for impossible values by looking at data ranges will test data quality. No post-treatment data will be released until the database is locked. Access to the final trial dataset will be available to the PIs and research assistant. The datasets generated and/or analysed during the current study will be included in the subsequent results publication. Anonymised participant level data available on request.

Data analysis

The data will be analysed using password protected NHS computers at NELFT. It will be analysed by PIs, the research worker and clinical psychology trainees as part of their doctoral theses. Thematic analysis of acceptability interviews will be completed before quantitative analysis of acceptability data to avoid bias. Dependent t-tests (or non-parametric equivalents) and effect sizes (Cohen's d) will be calculated for all outcomes measures. EDEQ change will be utilised in a power analysis to estimate sample size required for effectiveness trial. We will model for missing data of anybody who completed therapy.

For peer review only - http://bmjopen.bmj.com/site/about/guidelines.xhtml

Methods: Monitoring

Data Monitoring

A Research Steering Committee (RSG) comprised of the research team including CI and PIs, representatives from the sponsors, representatives from both EDS and PPI members (with history of anorexia and family members) oversees the SPEAKS research programme. It is chaired by an experienced researcher independent from trial Trusts and sponsor. The RSG meets every six months for reporting and discussion. Additional meetings can be convened at short notice if required. The RSG chair is available for independent advice to the research team.

Patient and Public Involvement

PPI has been in place since the inception of the study before trial funding was sought. An initial Research Design Service PPI Grant covered costs of a consultation series to assess SPEAKS model and trial validity. This informed intervention development, study design, and the SPEAKS name. Following trial funding, feedback on key documents such as the plain English summary and participant information sheets (PIS) were obtained via contacts made in this initial consultation. Further, PPI input continues throughout the study via the RSG and in open feedback events at key stages of the study advertised via local charity and support group networks.

Adverse event reporting and harms

Protocol violations such as hospital admission are recorded as per Trust guidance, and logged in trial records for reporting. Participants admitted to hospital will be withdrawn from the study. All research staff are NIHR Good Clinical Practice (GCP) trained and follow these guidelines for safety reporting procedures. Due to the feasibility design, there are no interim analyses or stopping guidelines; however, therapists monitor participant safety with regular risk assessments and communicate to the research team. Events leading to participants or others experiencing potential or actual serious harm are recorded by the researcher and reported to the PI and sponsor within 24

For peer review only - http://bmjopen.bmj.com/site/about/guidelines.xhtml

hours of knowledge of the event. Decision on expectedness and relatedness to the study intervention will be taken, with further investigation as required. The PI and sponsor will monitor events in case a pattern emerges, taking action if necessary. Clinical risk will be managed within ED service guidelines. Following completion of trial participation, all usual clinical service and NHS Trust standard operating procedures continue to apply with post-trial care continuing if clinically indicated. During the trial all usual Trust complaint procedures can be followed.

Auditing

Overall study conduct, and conduct at individual sites, will be monitored by the Sponsor Monitor at regular intervals. The Trial Master File (TMF) will be audited at site initiation, annually for the duration of the study and at study closure. This audit will follow the sponsor Standard Operating Procedures (SOP) and includes checks of: completeness and secure storage of TMF, study-wide approvals, study-wide safety and deviation/violation reporting, GCP compliance and performance of study against recruitment targets.

Individual research sites will be monitored after five participants have been recruited as part of site initiation, annually from site initiation date and at study closure at site. Monitoring will follow the sponsor SOP and include monitoring of the Investigator Site File (ISF) for completeness and secure storage, SDV checks, CRF checks, Serious Adverse Events and deviation/violation checks, GCP compliance and performance of site against recruitment targets.

Ethics and dissemination

Research ethics approval

This study has been reviewed in accordance with the guidelines for Canterbury Christ Church University research and has been approved by the London – Bromley Research Ethics Committee (NHS Rec Reference REC Ref: 19/LO/1530).

For peer review only

Protocol amendments

Any protocol amendments will be communicated to all relevant parties and research sites. Notice of intention to submit an amendment will be provided via email to the sponsor and the research governance representatives at all sites to offer an opportunity to discuss any queries prior to submission and confirm support. Ethical approval will subsequently be sought via online submission following IRAS guidance. Once ethical approval has been granted for an amendment this will be disseminated to all parties and official registries (such as ISRCTN) will be notified.

Consent

Patients meeting inclusion criteria will be provided with a PIS by their assessing clinician if they are willing to hear more about the study. Informed consent will be obtained at a face to face meeting with a member of the research team. The meeting will take place at least 48 hours after the participant has been provided with the PIS, with additional time given as necessary.

At the consent appointment, the research worker will answer any initial questions from the potential participant. They will review the PIS, highlighting key aspects and checking the patient's understanding of what study involvement entails. The researcher will explicitly state and make clear that deciding not to participate will not affect the patient's care in any way, and that if they decide to take part they can change their mind at any time without affecting their current or future care. The research team (like all other clinical staff members) will be trained in the principles of mental capacity and will hold this in mind throughout. Obtaining consent will be a focus of research supervision.

Confidentiality

In line with usual clinical practice, all participant information will be kept confidential, except as governed by law (i.e. if there is a legal obligation on the researcher to disclose this information to authorities due to risk concerns).

For peer review only - http://bmjopen.bmj.com/site/about/guidelines.xhtml

Signed informed consent forms will be stored separately from completed questionnaires and interview transcripts, which will be notated with only a participant number and/or pseudonym. Signed informed consent forms will be kept in a locked cabinet on NELFT or SPFT Property as relevant. Completed questionnaire data will be entered into study databases in encrypted Trust network folders accessible only to the research team. Any video recordings of therapy sessions and formulation descriptions will be stored on secure encrypted password protected NHS hard drives.

Participant identity will not be included in written interview transcripts, and will not be revealed in any publication resulting from this study. Data gathered from this study will be retained as required by regulations, which is up to ten years following publication of empirical articles or communications describing study results.

Availability of data and materials

Study databases and TMFs will only be available to the research team employed by the participating Trusts. The trainee psychologists analysing video recordings and psychological formulation data will not know participant identity beyond what they see in recorded therapy sessions. Qualitative interview recordings may be transcribed by a third-party agency approved by the sponsor. Participant names and personal details will not be disclosed and full confidentiality agreements will be established as per this protocol.

Dissemination policy

Data will be disseminated via high-impact, peer-reviewed journals, with Open Access sought where possible. Papers will be submitted for conference presentations to achieve dissemination to practitioners and professionals within the ED field. Dissemination to service users and clinical networks is considered extremely important and be achieved via service user networks across NHS Trusts and HEIs involved in the study, via charity networks (e.g. UK eating disorder charity BEAT) and

For peer review only - http://bmjopen.bmj.com/site/about/guidelines.xhtml

through a free open conference hosted by the NHS Trusts and HEI to disseminate findings and facilitate feedback on the SPEAKS clinical model and intervention. The decisions of when and where to publish data and authorship eligibility is decided by the SPEAKS RSG. Professional writers will not be used.

Discussion

This SPEAKS feasibility trial aims to assess acceptability, research and recruitment, sample size estimation and initial economic indications of the SPEAKS intervention for adults with AN Otherwise Specified Feeding or Eating Disorder-AN type. It also tests the hypothesised change process of the SPEAKS intervention.

Potential implications

This first test of the SPEAKS intervention will provide initial indication of the feasibility of SPEAKS and inform whether progression to larger trials is appropriate. Change process analyses will provide valuable insights into SPEAKS hypotheses, informing refinement of the intervention, whilst also contributing to broader evidence-base of relevant change processes for adults with AN.

Strengths

This feasibility trial is strengthened by multi-site design. Furthermore, sites included are relatively research naïve in delivery of clinical trials facilitating realistic insights into the feasibility of intervention delivery within naturalistic NHS services. Inclusion of consecutive referrals enables broad spectrum of participants across AN and OSFED severity and presentations, increasing generalisability. Robust measures to facilitate adherence to the model are included.

Challenges

For peer review only - http://bmjopen.bmj.com/site/about/guidelines.xhtml

SPEAKS is an experiential, relational model, designed for face-to-face settings. This trial began in the wake of the COVID-19 pandemic resulting in therapy sessions moving indefinitely to online video platforms with most participants never meeting their therapist in person. People with AN are often regarded as difficult to engage and have high levels of therapy dropout, thus issues of engagement with clinical and research protocols are a challenge even without this context. These factors may affect outcomes or length of delivery.

For peer review only

For peer review only - http://bmjopen.bmj.com/site/about/guidelines.xhtml

Declaration of competing interests

Not applicable. The authors confirm there are no interests to declare.

Funding

This feasibility study is funded as part of an Integrated Clinical Academic Fellowship-Clinical Lectureship awarded to AO (ICA-CL-2015-01-005) supported by the National Institute for Health Research and Health Education England. The views expressed in this publication are those of the authors and not necessarily those of the NHS, the National Institute for Health Research, Health Education England or the Department of Health.

For peer review only

Roles and Responsibilities

Author affiliation & Contributions

AO is affiliated with Kent and Medway All Age Eating Disorder Service, North East London NHS Foundation Trust (NELFT), The Courtyard, Pudding Lane, Maidstone, ME14 1PA, United Kingdom and The Salomons Institute for Applied Psychology, Canterbury Christ Church University, Canterbury, United Kingdom. Email: anna.oldershaw@nelft.nhs.uk Tel: 0300 300 1980

AO is the Chief Investigator and Corresponding Author. She led protocol development and wrote all trial documents. She is co-developer of the SPEAKS intervention, and wrote the SPEAKS intervention guidebook. She is Principal Investigator (PI) for the Kent site.

TL is affiliated with The Salomons Institute for Applied Psychology, Canterbury Christ Church University, Canterbury, United Kingdom. Tony.Lavender@canterbury.ac.uk

TL is co-developer of the SPEAKS intervention and offered feedback and edits on the SPEAKS intervention guidebook. He contributed to the study design and development of the protocol. He is chair of the RSG.

RB is affiliated with Kent and Medway All Age Eating Disorder Service NELFT.

Randeep.Basra@nelft.nhs.uk

RB is research assistant on the SPEAKS feasibility trial. He has provided input into the protocol and assisted in the preparation and content of this manuscript.

HS is affiliated with Sussex Eating Disorders Service and Research and Development Department, Sussex Partnership NHS Foundation Trust (SPFT), Sussex, United Kingdom.

Helen.Startup@sussexpartnership.nhs.uk HS is co-developer of the SPEAKS intervention and provided content, feedback and edits on the SPEAKS intervention guidebook. She contributed to the study design and development of the protocol. She is PI for the Sussex site.

All authors read and approved the final manuscript.

For peer review only - http://bmjopen.bmj.com/site/about/guidelines.xhtml

List of Figures

Figure 1. SPEAKS emotion change process

Figure 2. SPEAKS Treatment Phases

Figure 3. SPEAKS spirit schedule

Figure 4. SPEAKS Consort Diagram

References

1. Smink FR, Van Hoeken D, Hoek HW. Epidemiology of eating disorders: incidence, prevalence and mortality rates. Current psychiatry reports. 2012 Aug;14(4):406-14.
2. PricewaterhouseCoopers. The costs of eating disorders: Social, health and economic impacts. 2015. https://www.b-eat.co.uk/assets/000/000/302/The_costs_of_eating_disorders_Final_original.pdf Accessed 10 Dec, 2020.
3. Viana MC, Gruber MJ, Shahly V, Alhamzawi A, Alonso J, Andrade LH, Angermeyer MC, Benjet C, Bruffaerts R, Caldas-de-Almeida JM, Girolamo GD. Family burden related to mental and physical disorders in the world: results from the WHO World Mental Health (WMH) surveys. Brazilian Journal of Psychiatry. 2013 Jun;35(2):115-25.
4. National Institute of Health and Care Excellence Guideline. Eating disorders: recognition and treatment. NG69. London: National Institute of Health and Care Excellence. 2017.
5. Carter FA, Jordan J, McIntosh VV, Luty SE, McKenzie JM, Frampton CM, Bulik CM, Joyce PR. The long-term efficacy of three psychotherapies for anorexia nervosa: A randomized, controlled trial. International Journal of eating disorders. 2011 Nov;44(7):647-54.
6. Fairburn CG, Bailey-Straebler S, Basden S, Doll HA, Jones R, Murphy R, O'Connor ME, Cooper Z. A transdiagnostic comparison of enhanced cognitive behaviour therapy (CBT-E) and

For peer review only - http://bmjopen.bmj.com/site/about/guidelines.xhtml

interpersonal psychotherapy in the treatment of eating disorders. Behaviour research and therapy. 2015 Jul 1;70:64-71.

7. Schmidt, U., Oldershaw, A., Jichi, F., Sternheim, L., Startup, H., McIntosh, V., ... & Landau, S. (2012). Out-patient psychological therapies for adults with anorexia nervosa: randomised controlled trial. *The British Journal of Psychiatry*, *201*(5), 392-399.

8. Schmidt U, Magill N, Renwick B, Keyes A, Kenyon M, Dejong H, Lose A, Broadbent H, Loomes R, Yasin H, Watson C. The Maudsley Outpatient Study of Treatments for Anorexia Nervosa and Related Conditions (MOSAIC): Comparison of the Maudsley Model of Anorexia Nervosa Treatment for Adults (MANTRA) with specialist supportive clinical management (SSCM) in outpatients with broadly defined anorexia nervosa: A randomized controlled trial. Journal of consulting and clinical psychology. 2015 Aug;83(4):796.

9. Zipfel S, Wild B, Groß G, Friederich HC, Teufel M, Schellberg D, Giel KE, de Zwaan M, Dinkel A, Herpertz S, Burgmer M. Focal psychodynamic therapy, cognitive behaviour therapy, and optimised treatment as usual in outpatients with anorexia nervosa (ANTOP study): randomised controlled trial. The Lancet. 2014 Jan 11;383(9912):127-37.

10. DeJong H, Broadbent H, Schmidt U. A systematic review of dropout from treatment in outpatients with anorexia nervosa. International Journal of Eating Disorders. 2012 Jul;45(5):635-47.

11. Bulik CM. The challenges of treating anorexia nervosa. Lancet (London, England). 2014 Jan 11;383(9912):105.

12. Startup H, Mountford V, Lavender A, Schmidt U. Cognitive behavioural case formulation in complex eating disorders. Case formulation in cognitive behaviour therapy: The treatment of challenging and complex cases. 2015 Jul 6:239-64.

13. Kass AE, Kolko RP, Wilfley DE. Psychological treatments for eating disorders. Current opinion in psychiatry. 2013 Nov;26(6):549.

For peer review only

For peer review only - http://bmjopen.bmj.com/site/about/guidelines.xhtml

14. Sala M, Heard A, Black EA. Emotion-focused treatments for anorexia nervosa: a systematic review of the literature. Eating and Weight Disorders-Studies on Anorexia, Bulimia and Obesity. 2016 Jun 1;21(2):147-64.
15. Oldershaw A, Lavender T, Schmidt U. Are socio-emotional and neurocognitive functioning predictors of therapeutic outcomes for adults with anorexia nervosa?. European Eating Disorders Review. 2018 Jul;26(4):346-59.
16. Kendler KS, Campbell J. Interventionist causal models in psychiatry: repositioning the mind-body problem. Psychological medicine. 2009 Jun 1;39(6):881.
17. Oldershaw A, Startup H, Lavender T. Anorexia Nervosa and a lost emotional self: A psychological formulation of the development, maintenance, and treatment of Anorexia Nervosa. Frontiers in Psychology. 2019 Mar 4;10:219.
18. Oldershaw A, Startup H. 18 Building the Healthy Adult in eating disorders. Creative Methods in Schema Therapy: Advances and Innovation in Clinical Practice. 2020 Jun 18.
19. Royal College of Psychiatrists, Royal College of Pathologists, Royal College of Physicians. *MARSIPAN: Management of Really Sick Patients with Anorexia Nervosa, 2nd edition* (College Report CR189). Royal College of Psychiatrists.
20. Craig P, Dieppe P, Macintyre S, Michie S, Nazareth I, Petticrew M. Developing and evaluating complex interventions: the new Medical Research Council guidance. Bmj. 2008 Sep 29;337.
21. Carroll KM, Nuro KF. One size cannot fit all: A stage model for psychotherapy manual development. Clinical Psychology: Science and Practice. 2002 Dec;9(4):396-406.
22. Elliott, R., & Rodgers, B. (2008). Client Change Interview Schedule (v5). University of Strathclyde, Glasgow.
23. Borelli B, Sepinwall D, Ernst D, Bellg AJ, Czajkowski S, Breger R, DeFrancesco C, Levesque C, Sharp DL, Ogedegbe G, Resnick B. A new tool to assess treatment fidelity and evaluation of treatment fidelity across 10 years of health behavior research. Journal of consulting and clinical psychology. 2005 Oct;73(5):852.

For peer review only - http://bmjopen.bmj.com/site/about/guidelines.xhtml

24. Decker SE, Nich C, Carroll KM, Martino S. Development of the therapist empathy scale. Behavioural and cognitive psychotherapy. 2014 May;42(3):339-54.

25. Fairburn CG, Beglin SJ. Eating disorder examination questionnaire. Cognitive behavior therapy and eating disorders. 2008 Sep:309-13.

26. Henry JD, Crawford JR. The short-form version of the Depression Anxiety Stress Scales (DASS-21): Construct validity and normative data in a large non-clinical sample. British journal of clinical psychology. 2005 Jun;44(2):227-39.

27. Bohn K, Fairburn CG. The clinical impairment assessment questionnaire (CIA). Cognitive behavioral therapy for eating disorders. 2008:1-3.

28. Manser R, Cooper M, Trefusis J. Beliefs about emotions as a metacognitive construct: Initial development of a self-report questionnaire measure and preliminary investigation in relation to emotion regulation. Clinical Psychology & Psychotherapy. 2012 May;19(3):235-46.

29. Young JE, Brown G. Young Schema Questionnaire-Short Form; Version 3. Psychological Assessment. 2005.

30. Simpson SG, Pietrabissa G, Rossi A, Seychell T, Manzoni GM, Munro C, Nesci JB, Castelnuovo G. Factorial structure and preliminary validation of the Schema Mode Inventory for Eating Disorders (SMI-Ed). Frontiers in psychology. 2018 Apr 24;9:600.

31. Jack DC. The silencing the self scale. Fairhaven Faculty Publications. 2017.

32. Tapal A, Oren E, Dar R, Eitam B. The sense of agency scale: A measure of consciously perceived control over one's mind, body, and the immediate environment. Frontiers in psychology. 2017 Sep 12;8:1552.

33. Gratz KL, Roemer L. Multidimensional assessment of emotion regulation and dysregulation: Development, factor structure, and initial validation of the difficulties in emotion regulation scale. Journal of psychopathology and behavioral assessment. 2004 Mar 1;26(1):41-54.

For peer review only - http://bmjopen.bmj.com/site/about/guidelines.xhtml

34. Chisholm D, Knapp MR, Knudsen HC, Amaddeo F, Gaite LU, Van Wijngaarden BO, EPSILON Study Group. Client socio-demographic and service receipt inventory–European version: development of an instrument for international research: EPSILON Study 5. The British Journal of Psychiatry. 2000 Jul;177(S39):s28-33.

35. Gonçalves MM, Ribeiro AP, Mendes I, Matos M, Santos A. Tracking novelties in psychotherapy process research: The innovative moments coding system. Psychotherapy research. 2011 Sep 1;21(5):497-509.

36. Pascual-Leone A, Greenberg LS. Classification of affective-meaning states. A. Pascual-Leone, Emotional processing in the therapeutic hour: Why “the only way out is through. 2005:289-367.

37. Billingham SA, Whitehead AL, Julious SA. An audit of sample sizes for pilot and feasibility trials being undertaken in the United Kingdom registered in the United Kingdom Clinical Research Network database. BMC medical research methodology. 2013 Dec;13(1):1-6.

38. Teare MD, Dimairo M, Shephard N, Hayman A, Whitehead A, Walters SJ. Sample size requirements to estimate key design parameters from external pilot randomised controlled trials: a simulation study. Trials. 2014 Dec;15(1):1-3.

For peer review only

For peer review only - http://bmjopen.bmj.com/site/about/guidelines.xhtml

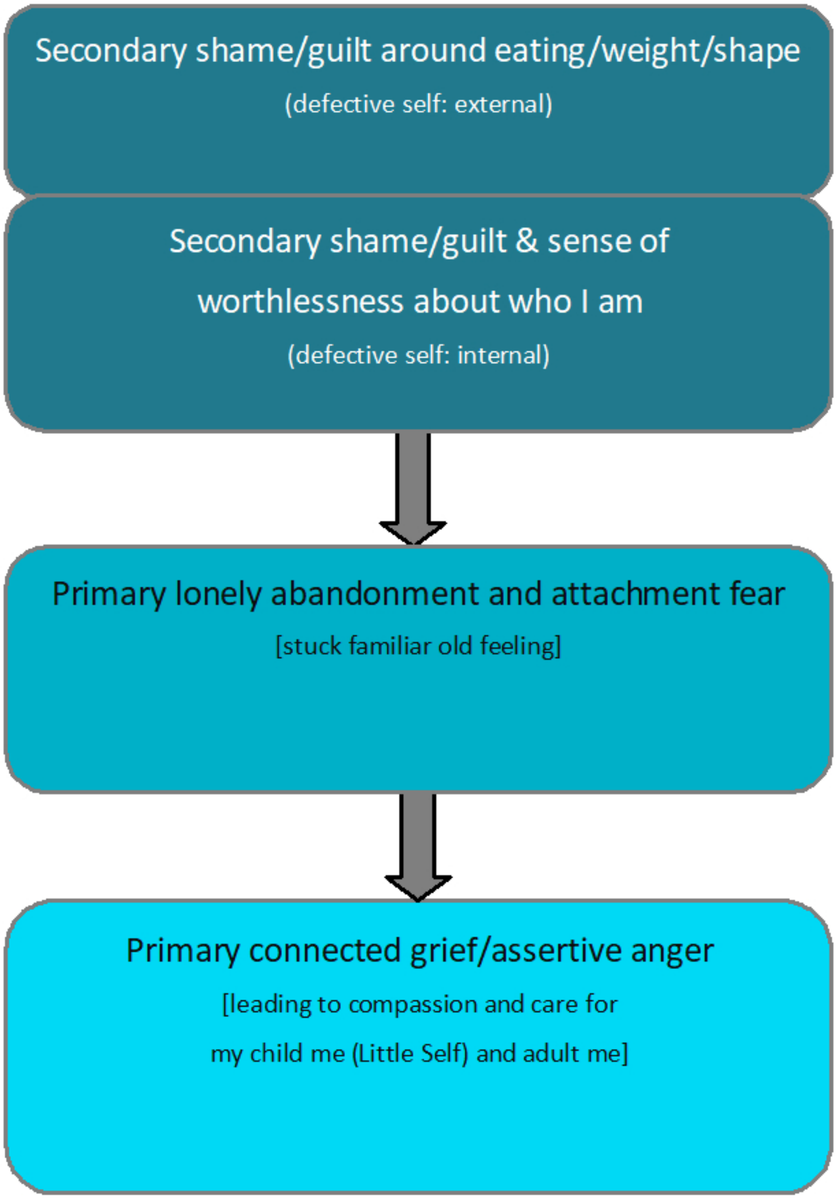

Figure 1. SPEAKS hypothesised change process

330x472mm (47 x 47 DPI)

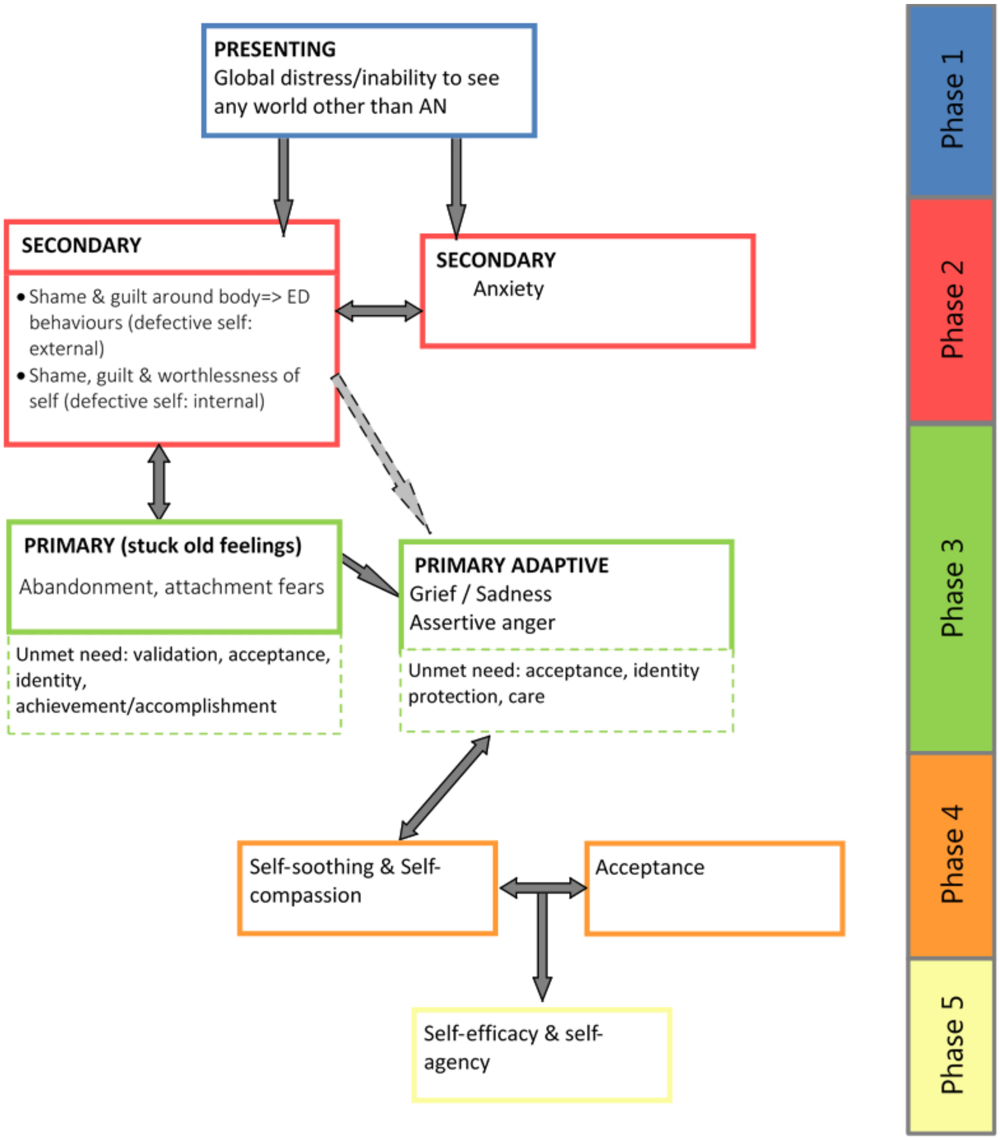

Figure 2. SPEAKS treatment phases

424x480mm (47 x 47 DPI)

		SPEAKS STUDY PERIOD				
	Enrolment	Post-enrolment				Post-intervention
TIMEPOINT	$-t_1$	Baseline	3 months	6 months	9 months	12-15 months
ENROLMENT:						
Eligibility screen	X					
Informed consent	X					
INTERVENTION:						
SPEAKS ASSESSMENTS						
Adapted CSSRI		X				X
Clinical outcome measures		X	X	X	X	X
Intervention-specific measures		X	X	X	X	X
Qualitative interviews						X

Figure 3. Spirit feasibility trial schedule

384x358mm (59 x 59 DPI)

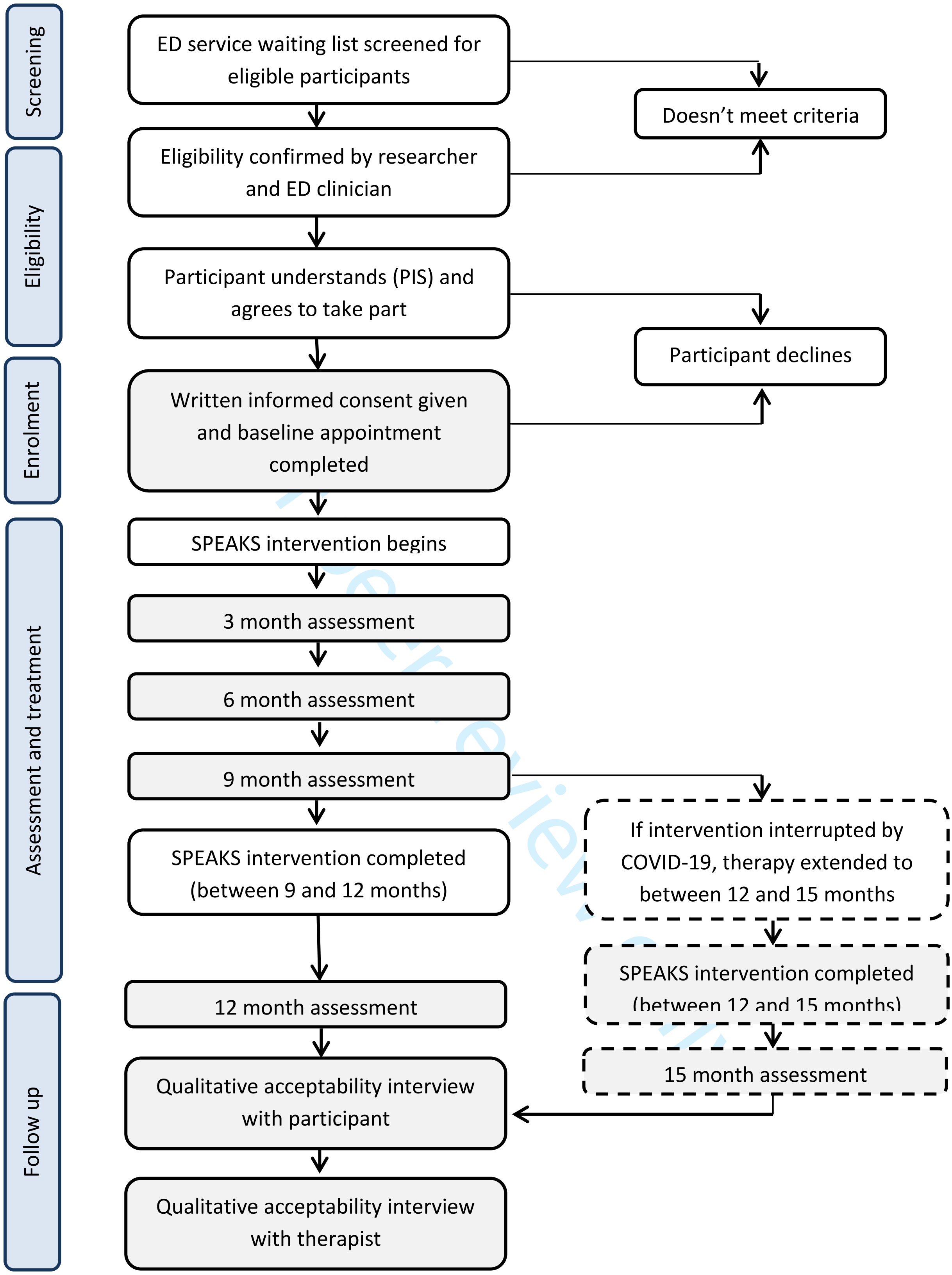
Screening
Eligibility
Enrolment
Assessment and treatment
Follow up
ED service waiting list screened for eligible participants
Doesn't meet criteria
Eligibility confirmed by researcher and ED clinician
Participant understands (PIS) and agrees to take part
Participant declines
Written informed consent given and baseline appointment completed
SPEAKS intervention begins
3 month assessment
6 month assessment
9 month assessment
If intervention interrupted by COVID-19, therapy extended to between 12 and 15 months
SPEAKS intervention completed (between 9 and 12 months)
SPEAKS intervention completed (between 12 and 15 months)
12 month assessment
15 month assessment
Qualitative acceptability interview with participant
Qualitative acceptability interview with therapist

SPIRIT

STANDARD PROTOCOL ITEMS: RECOMMENDATIONS FOR INTERVENTIONAL TRIALS

SPIRIT 2013 Checklist: Recommended items to address in a clinical trial protocol and related documents* – completed for SPEAKS Feasibility Trial protocol

Section/item	ItemNo	Description	Addressed on page(s)
**Administrative information**			
Title	1	Descriptive title identifying the study design, population, interventions, and, if applicable, trial acronym	1
Trial registration	2a	Trial identifier and registry name. If not yet registered, name of intended registry	4
	2b	All items from the World Health Organization Trial Registration Data Set	Available in trial registry
Protocol version	3	Date and version identifier	4
Funding	4	Sources and types of financial, material, and other support	22
Roles and responsibilities	5a	Names, affiliations, and roles of protocol contributors	22
	5b	Name and contact information for the trial sponsor	4
	5c	Role of study sponsor and funders, if any, in study design; collection, management, analysis, and interpretation of data; writing of the report; and the decision to submit the report for publication, including whether they will have ultimate authority over any of these activities	16-19
	5d	Composition, roles, and responsibilities of the coordinating centre, steering committee, endpoint adjudication committee, data management team, and other individuals or groups overseeing the trial, if applicable (see Item 21a for data monitoring committee)	16

For peer review only - http://bmjopen.bmj.com/site/about/guidelines.xhtml

**Introduction**			
Background and rationale	6a	Description of research question and justification for undertaking the trial, including summary of relevant studies (published and unpublished) examining benefits and harms for each intervention	5
	6b	Explanation for choice of comparators	n/a
Objectives	7	Specific objectives or hypotheses	6
Trial design	8	Description of trial design including type of trial (eg, parallel group, crossover, factorial, single group), allocation ratio, and framework (eg, superiority, equivalence, noninferiority, exploratory)	7
**Methods: Participants, interventions, and outcomes**			
Study setting	9	Description of study settings (eg, community clinic, academic hospital) and list of countries where data will be collected. Reference to where list of study sites can be obtained	8
Eligibility criteria	10	Inclusion and exclusion criteria for participants. If applicable, eligibility criteria for study centres and individuals who will perform the interventions (eg, surgeons, psychotherapists)	8
Interventions	11a	Interventions for each group with sufficient detail to allow replication, including how and when they will be administered	9
	11b	Criteria for discontinuing or modifying allocated interventions for a given trial participant (eg, drug dose change in response to harms, participant request, or improving/worsening disease)	9
	11c	Strategies to improve adherence to intervention protocols, and any procedures for monitoring adherence (eg, drug tablet return, laboratory tests)	10-12

For peer review only - http://bmjopen.bmj.com/site/about/guidelines.xhtml

	11d	Relevant concomitant care and interventions that are permitted or prohibited during the trial	9
Outcomes	12	Primary, secondary, and other outcomes, including the specific measurement variable (eg, systolic blood pressure), analysis metric (eg, change from baseline, final value, time to event), method of aggregation (eg, median, proportion), and time point for each outcome. Explanation of the clinical relevance of chosen efficacy and harm outcomes is strongly recommended	12-13
Participant timeline	13	Time schedule of enrolment, interventions (including any run-ins and washouts), assessments, and visits for participants. A schematic diagram is highly recommended (see Figure)	14
Sample size	14	Estimated number of participants needed to achieve study objectives and how it was determined, including clinical and statistical assumptions supporting any sample size calculations	14
Recruitment	15	Strategies for achieving adequate participant enrolment to reach target sample size	14
**Methods: Assignment of interventions (for controlled trials)**			
Allocation:			
Sequence generation	16a	Method of generating the allocation sequence (eg, computer-generated random numbers), and list of any factors for stratification. To reduce predictability of a random sequence, details of any planned restriction (eg, blocking) should be provided in a separate document that is unavailable to those who enrol participants or assign interventions	15

For peer review only - http://bmjopen.bmj.com/site/about/guidelines.xhtml

Allocation concealment mechanism	16b	Mechanism of implementing the allocation sequence (eg, central telephone; sequentially numbered, opaque, sealed envelopes), describing any steps to conceal the sequence until interventions are assigned	15
Implementation	16c	Who will generate the allocation sequence, who will enrol participants, and who will assign participants to interventions	15
Blinding (masking)	17a	Who will be blinded after assignment to interventions (eg, trial participants, care providers, outcome assessors, data analysts), and how	15
	17b	If blinded, circumstances under which unblinding is permissible, and procedure for revealing a participant's allocated intervention during the trial	n/a
**Methods: Data collection, management, and analysis**			
Data collection methods	18a	Plans for assessment and collection of outcome, baseline, and other trial data, including any related processes to promote data quality (eg, duplicate measurements, training of assessors) and a description of study instruments (eg, questionnaires, laboratory tests) along with their reliability and validity, if known. Reference to where data collection forms can be found, if not in the protocol	15
	18b	Plans to promote participant retention and complete follow-up, including list of any outcome data to be collected for participants who discontinue or deviate from intervention protocols	15
Data management	19	Plans for data entry, coding, security, and storage, including any related processes to promote data quality (eg, double data entry; range checks for data values). Reference to where details of data management procedures can be found, if not in the protocol	15

For peer review only - http://bmjopen.bmj.com/site/about/guidelines.xhtml

Statistical methods	20a	Statistical methods for analysing primary and secondary outcomes. Reference to where other details of the statistical analysis plan can be found, if not in the protocol	15-16
	20b	Methods for any additional analyses (eg, subgroup and adjusted analyses)	15-16
	20c	Definition of analysis population relating to protocol non-adherence (eg, as randomised analysis), and any statistical methods to handle missing data (eg, multiple imputation)	15-16
**Methods: Monitoring**			
Data monitoring	21a	Composition of data monitoring committee (DMC); summary of its role and reporting structure; statement of whether it is independent from the sponsor and competing interests; and reference to where further details about its charter can be found, if not in the protocol. Alternatively, an explanation of why a DMC is not needed	16
	21b	Description of any interim analyses and stopping guidelines, including who will have access to these interim results and make the final decision to terminate the trial	16
Harms	22	Plans for collecting, assessing, reporting, and managing solicited and spontaneously reported adverse events and other unintended effects of trial interventions or trial conduct	16-17
Auditing	23	Frequency and procedures for auditing trial conduct, if any, and whether the process will be independent from investigators and the sponsor	17
**Ethics and dissemination**			
Research ethics approval	24	Plans for seeking research ethics committee/institutional review board (REC/IRB) approval	18

For peer review only - http://bmjopen.bmj.com/site/about/guidelines.xhtml

Protocol amendments	25	Plans for communicating important protocol modifications (eg, changes to eligibility criteria, outcomes, analyses) to relevant parties (eg, investigators, REC/IRBs, trial participants, trial registries, journals, regulators)	18
Consent or assent	26a	Who will obtain informed consent or assent from potential trial participants or authorised surrogates, and how (see Item 32)	18
	26b	Additional consent provisions for collection and use of participant data and biological specimens in ancillary studies, if applicable	n/a
Confidentiality	27	How personal information about potential and enrolled participants will be collected, shared, and maintained in order to protect confidentiality before, during, and after the trial	19
Declaration of interests	28	Financial and other competing interests for principal investigators for the overall trial and each study site	22
Access to data	29	Statement of who will have access to the final trial dataset, and disclosure of contractual agreements that limit such access for investigators	15
Ancillary and post-trial care	30	Provisions, if any, for ancillary and post-trial care, and for compensation to those who suffer harm from trial participation	17
Dissemination policy	31a	Plans for investigators and sponsor to communicate trial results to participants, healthcare professionals, the public, and other relevant groups (eg, via publication, reporting in results databases, or other data sharing arrangements), including any publication restrictions	20
	31b	Authorship eligibility guidelines and any intended use of professional writers	20

For peer review only

	31c	Plans, if any, for granting public access to the full protocol, participant-level dataset, and statistical code	15
**Appendices**			
Informed consent materials	32	Model consent form and other related documentation given to participants and authorised surrogates	Appendix
Biological specimens	33	Plans for collection, laboratory evaluation, and storage of biological specimens for genetic or molecular analysis in the current trial and for future use in ancillary studies, if applicable	n/a

*It is strongly recommended that this checklist be read in conjunction with the SPIRIT 2013 Explanation & Elaboration for important clarification on the items. Amendments to the protocol should be tracked and dated. The SPIRIT checklist is copyrighted by the SPIRIT Group under the Creative Commons "Attribution-NonCommercial-NoDerivs 3.0 Unported" license.

For peer review only

For peer review only - http://bmjopen.bmj.com/site/about/guidelines.xhtml

TIDieR
Template for Intervention Description and Replication

The TIDieR (Template for Intervention Description and Replication) Checklist*:

Information to include when describing an intervention and the location of the information

Item number	Item	Where located ** Primary paper (page or appendix number)	Other † (details)
	**BRIEF NAME**		
**1.**	Provide the name or a phrase that describes the intervention.	Title page 1	______________
	**WHY**		
**2.**	Describe any rationale, theory, or goal of the elements essential to the intervention.	Intro page 5	______________
	**WHAT**		
**3.**	Materials: Describe any physical or informational materials used in the intervention, including those provided to participants or used in intervention delivery or in training of intervention providers. Provide information on where the materials can be accessed (e.g. online appendix, URL).	page 10	______________
**4.**	Procedures: Describe each of the procedures, activities, and/or processes used in the intervention, including any enabling or support activities.	page 10	______________
	**WHO PROVIDED**		
**5.**	For each category of intervention provider (e.g. psychologist, nursing assistant), describe their expertise, background and any specific training given.	page 9	______________
	**HOW**		
**6.**	Describe the modes of delivery (e.g. face-to-face or by some other mechanism, such as internet or telephone) of the intervention and whether it was provided individually or in a group.	page 9	______________
	**WHERE**		
**7.**	Describe the type(s) of location(s) where the intervention occurred, including any necessary infrastructure or relevant features.	page 9	______________

For peer review only - http://bmjopen.bmj.com/site/about/guidelines.xhtml

	**WHEN and HOW MUCH**		
**8.**	Describe the number of times the intervention was delivered and over what period of time including the number of sessions, their schedule, and their duration, intensity or dose.	page 9	______
	**TAILORING**		
**9.**	If the intervention was planned to be personalised, titrated or adapted, then describe what, why, when, and how.	page 9	______
	**MODIFICATIONS**		
**10.**‡	If the intervention was modified during the course of the study, describe the changes (what, why, when, and how).	n/a	______
	**HOW WELL**		
**11.**	Planned: If intervention adherence or fidelity was assessed, describe how and by whom, and if any strategies were used to maintain or improve fidelity, describe them.	page 11-12	______
**12.**‡	Actual: If intervention adherence or fidelity was assessed, describe the extent to which the intervention was delivered as planned.	n/a	______

** **Authors** - use N/A if an item is not applicable for the intervention being described. **Reviewers** – use '?' if information about the element is not reported/not sufficiently reported.

† If the information is not provided in the primary paper, give details of where this information is available. This may include locations such as a published protocol or other published papers (provide citation details) or a website (provide the URL).

‡ If completing the TIDieR checklist for a protocol, these items are not relevant to the protocol and cannot be described until the study is complete.

* We strongly recommend using this checklist in conjunction with the TIDieR guide (see *BMJ* 2014;348:g1687) which contains an explanation and elaboration for each item.

* The focus of TIDieR is on reporting details of the intervention elements (and where relevant, comparison elements) of a study. Other elements and methodological features of studies are covered by other reporting statements and checklists and have not been duplicated as part of the TIDieR checklist. When a **randomised trial** is being reported, the TIDieR checklist should be used in conjunction with the CONSORT statement (see www.consort-statement.org) as an extension of **Item 5 of the CONSORT 2010 Statement.** When a **clinical trial protocol** is being reported, the TIDieR checklist should be used in conjunction with the SPIRIT statement as an extension of **Item 11 of the SPIRIT 2013 Statement** (see www.spirit-statement.org). For alternate study designs, TIDieR can be used in conjunction with the appropriate checklist for that study design (see www.equator-network.org).

For peer review only - http://bmjopen.bmj.com/site/about/guidelines.xhtml
For peer review only

BMJ Open

The SPEAKS study: Study protocol of a multisite feasibility trial of the Specialist Psychotherapy with Emotion for Anorexia in Kent and Sussex (SPEAKS) intervention for outpatients with anorexia nervosa or otherwise specified feeding and eating disorders, anorexia nervosa type

Journal:	*BMJ Open*
Manuscript ID	bmjopen-2021-050350.R2
Article Type:	Protocol
Date Submitted by the Author:	08-Dec-2021

Complete List of Authors:	Oldershaw, Anna; North East London NHS Foundation Trust Goodmayes Hospital, Kent and Medway All Age Eating Disorder Service; Canterbury Christ Church University Faculty of Social and Applied Sciences, Salomons Institute for Applied Psychology<br>Lavender, Tony; Canterbury Christ Church University, Salomons Institute for Applied Psychology<br>Basra, Randeep; North East London NHS Foundation Trust Goodmayes Hospital, Kent and Medway All Age Eating Disorder Service<br>Startup, Helen; Sussex Partnership NHS Foundation Trust, Brighton and Hove Eating Disorder Service

**Primary Subject Heading**:	Mental health
Secondary Subject Heading:	Evidence based practice, Research methods
Keywords:	Eating disorders < PSYCHIATRY, Adult psychiatry < PSYCHIATRY, QUALITATIVE RESEARCH

SCHOLARONE™
Manuscripts

The SPEAKS study: Study protocol of a multisite feasibility trial of the Specialist Psychotherapy with Emotion for Anorexia in Kent and Sussex (SPEAKS) intervention for outpatients with anorexia nervosa or otherwise specified feeding and eating disorders, anorexia nervosa type

Anna Oldershaw*

Kent and Medway All Age Eating Disorder Service, North East London NHS Foundation Trust (NELFT), The Courtyard, Pudding Lane, Maidstone, ME14 1PA, United Kingdom and The Salomons Institute for Applied Psychology, Canterbury Christ Church University, Canterbury, United Kingdom.

Email: anna.oldershaw@nelft.nhs.uk Tel: 0300 300 1980 *Corresponding author

Tony Lavender

The Salomons Institute for Applied Psychology, Canterbury Christ Church University, Canterbury, United Kingdom.

Randeep Basra

Kent and Medway All Age Eating Disorder Service North East London NHS Foundation Trust (NELFT), Maidstone, United Kingdom.

Helen Startup

Sussex Eating Disorders Service and Research and Development Department, Sussex Partnership NHS Foundation Trust (SPFT), Sussex, United Kingdom.

Keywords: Anorexia nervosa, eating disorders, emotions, emotion focused therapy, schema therapy

Word Count: 3999

Abstract

For peer review only - http://bmjopen.bmj.com/site/about/guidelines.xhtml

Introduction

Anorexia nervosa (AN) is a severe mental health condition associated with high mortality rates and significantly impaired quality of life. National guidelines outline psychotherapeutic interventions as treatments of choice for adults with AN, but outcomes are limited and therapy drop-out high, resulting in calls for new innovative treatments. The Specialist Psychotherapy with Emotion for Anorexia in Kent and Sussex (SPEAKS) research programme sought to develop the SPEAKS intervention avoiding some difficulties inherent in development of earlier interventions, such unclear hypotheses about change processes. SPEAKS focuses on a core hypothesized maintaining factor (emotional experience) with clear proposed model of change. The current feasibility trial aims to provide an initial test of SPEAKS and inform design of a full randomised controlled trial protocol.

Methods & Analysis

This study employs a multi-site, single-arm, within-group, mixed-methods design. Up to 60 participants (36 therapy completers) meeting inclusion criteria will be offered the SPEAKS intervention instead of treatment-as-usual (TAU). SPEAKS is a weekly psychotherapy lasting nine to 12 months, provided by trained and experienced eating disorders therapists. All other clinical input remains inline with TAU. *Acceptability* will be assessed using VAS scales and end of therapy interview. *Reach and recruitment*, such as recruitment yield, will be monitored. To support *sample size estimation* and *economic estimation*, data pertaining to eating disorder-related symptoms will be recorded every three months, alongside service usage and intervention-specific measures. Videoed therapy sessions will inform model adherence. Additional analyses coding videoed therapy will test SPEAKS change process hypotheses.

Ethics & Dissemination

Ethical approval has been granted by London–Bromley Research Ethics Committee (NHS Rec Reference: 19/LO/1530). Data will be disseminated via high-impact, peer-reviewed journals (Open

For peer review only - http://bmjopen.bmj.com/site/about/guidelines.xhtml
For peer review only

Access preferred), conferences, service user and charity networks (e.g. UK charity BEAT) and through a free open conference hosted by NHS Trusts and Higher Education Institutions (HEI).

Article summary: Strengths and Limitations

- This feasibility trial studies a novel psychotherapy for adults with AN, with a focus on emotional change: The SPEAKS intervention.
- This feasibility study is strengthened by its multi-site trial design, completed in largely 'research naïve' NHS services, increasing insight into its feasibility and generalisability.
- The study is limited by its single arm design and non-blinded procedures, which may result in biases such as allegiance effects and does not allow for estimating acceptability of a randomised design.
- In addition to its feasibility aims regarding the SPEAKS intervention, the trial design includes analysis of the therapeutic change process with wider implications for understanding therapeutic change for adults with AN.
- The trial has been subject to the challenges imposed by the COVID-19 pandemic including regarding clinical and research engagement.

For peer review only

For peer review only - http://bmjopen.bmj.com/site/about/guidelines.xhtml

Trial registration

Registered on ISRCTN trials registry reference ISRCTN11778891. Prospectively applied 28th November 2019; retrospectively assigned 20th January 2020.

http://www.isrctn.com/ISRCTN11778891

Trial Status

Recruitment began on 12th December 2019 and ends 28th February 2021. All data will be collected and the trial ended by 28th February 2022.

Protocol version

SPEAKS protocol version 3.0 (30/08/2020). Changed were made to the original protocol due to the COVID-19 pandemic. A further set of changes were made to incorporate the measures of change processes, resulting in this being the third version of the protocol.

Trial sponsor information

North East London NHS Foundation Trust

Research and Development Department

1st Floor, Maggie Lilley Suite

Goodmayes Hospital

Barley Lane

Ilford

IG3 8XJ

+44 (0)300 555 1200

Fiona.Horton@NELFT.nhs.uk

For peer review only - http://bmjopen.bmj.com/site/about/guidelines.xhtml

Introduction

Background and rationale

Anorexia nervosa (AN) is a severe mental illness with poor prognosis and the highest mortality rate of any psychiatric disorder [1]. Early intervention is key, but is impeded by a delay of around 18 months from symptoms emerging to treatment, and with multiple relapses likely[2]. Caregiving for somebody with severe AN is almost twice as lengthy as for somebody with a physical health disorder (e.g., cancer) or other mental health difficulty (e.g., psychosis)[3]. Thus significant costs exist financially and emotionally to the individual, family and carers, as well as society as a whole, offering a “compelling case for change” in services and treatment[2, p. 9].

Most recent NICE guidelines in the UK recommend that psychotherapeutic interventions be considered for adults with AN[4]. Yet results from randomized controlled trials (RCTs) indicate that out-patient treatments do not out-perform each other or control comparisons[5-9] with weight-restoration achieved by 20 percent of patients after one year[7-9]. AN is highly valued by sufferers resulting in poor treatment engagement and high dropout rates[10]. Thus there is an urgent need to develop innovative interventions that can engage people with AN[4, 11, 12], while targeting unique factors involved in its development and maintenance[13]. Emotional experience has long been recognised as a factor in the development and maintenance of AN and is recognised as a promising target area for therapies[14] and increasingly incorporated into psychotherapy interventions for adults with AN[5-9]. However, outcomes remain limited and it is unclear to what extent emotional difficulties are targeted by these interventions[15].

This trial comprises part of the SPEAKS programme which aimed to develop and test in a feasibility study an emotion focused intervention for adults with anorexia (the SPEAKS intervention). This research programme sought to overcome several key difficulties with the development and application of some earlier interventions, such as unsuccessful targeting of variables or lack of clarity

For peer review only - http://bmjopen.bmj.com/site/about/guidelines.xhtml

on how change is achieved. The SPEAKS programme proposes focussing on a core clearly defined model with one key putative maintaining process (emotional experience in AN) and following an 'interventionist-causal model approach'[16]. By developing a new model of the development and maintenance of AN drawn from integration of quantitative and qualitative research, it seeks to ensure an emotions' focus[17]. The SPEAKS intervention hypothesises a clear and testable change process to be targeted in therapy[18; Figure 1]; thus affording the ability to examine proposed mechanisms of change enabling further evidence-based development and refinement of the model. This body of work involved close partnering with stakeholders including those with current and past experience of AN, families, therapists and service managers.

Objectives

This multi-site feasibility trial aims to investigate the SPEAKS intervention in the following domains:

- Acceptability
- Reach and recruitment
- Adherence and compliance
- Sample size & economic evaluation to establish parameters and financial costs of a potential future efficacy/effectiveness trial
- Change process analysis

We hypothesise that:

(1) SPEAKS will be acceptable to participants and therapists.

(2) SPEAKS will meet sufficient reach and recruitment expectations to support progression to full effectiveness/efficacy trial.

(3) Therapists and service users will be able to adhere to the therapy model as intended and the research requirements

For peer review only - http://bmjopen.bmj.com/site/about/guidelines.xhtml

For peer review only

(4) Participant change over time will reflect the proposed emotion change process outlined in Figure 1.

Trial design

The SPEAKS study is a multi-site, single-armed, within-group mixed-methods design.

For peer review only

Methods: Participants, interventions and outcomes

Study setting

This feasibility study runs in two outpatient specialist eating disorders services (EDS) within the UK National Health Service (NHS): Kent & Medway All Age Eating Disorder Service at NELFT and Sussex Eating Disorder Service at SPFT.

Eligibility criteria

Service Users

Service users are eligible to participate if they:

(1) Are referred into Kent or Sussex EDS and meet service criteria (e.g. registered with a local GP).

(2) Meet Diagnostic and Statistical Manual 5 Criteria for Anorexia Nervosa or OSFED (Other Specified Feeding or Eating Disorder) of Anorexic type.

(3) Are aged 18 or above.

(4) Have BMI >15kg/m^2

(5) Have sufficient English language abilities to complete a talking therapy.

Service users are excluded if they have/are:

(1) Rated as 'High Risk', or as 'High Concern' in weight criteria, on the MARSIPAN Guidelines for adults with eating disorders (i.e. BMI<15; weight loss>500g for 2 consecutive weeks)[19]

(2) Considerable psychological risk, including active suicidal thoughts and plans.

(3) Comorbidity requiring treatment priority.

(4) Alcohol/substance use disorder.

(5) Participating in another treatment trial

(6) Diagnosed Intellectual disability impeding ability to utilise therapy

For peer review only - http://bmjopen.bmj.com/site/about/guidelines.xhtml

For peer review only

(7) Pregnant

Therapists

Therapists are eligible to offer SPEAKS in the trial if they:

(1) Are a specialist eating disorder therapist > three years experience

(2) Work in Kent All Age Eating Disorder Service or Sussex Eating Disorder Service

(3) Have specialist training in an experiential dialogical self chairwork model (e.g. emotion-focused therapy, schema therapy, compassion-focused therapy)

Interventions

SPEAKS Intervention

SPEAKS is an individual outpatient psychotherapy for adults with AN. Participants receive weekly individual sessions of psychotherapy for 9-12 months with two follow-up sessions within 3 months of completion. SPEAKS is intended to be offered face-to-face in a clinic setting; however, due to the COVID-19 pandemic video sessions via on an online platform are provided.

SPEAKS is a direct replacement for psychotherapy as usual. Therapists receive fortnightly supervision by supervisors trained in the supervision requirements of SPEAKS. The services at both sites are in the same region of the UK and follow national NHS treatment guidelines. All usual care procedures, including additional interventions such as dietician appointments or inpatient referrals will remain the same, but be reported. People will be removed from the trial if they require immediate inpatient treatment at any point or request removal for any reason, and these data will be reported.

Intervention Development. The development of SPEAKS is consistent with MRC guidance for complex interventions[20]. SPEAKS was developed following a clear programme of research to integrate quantitative and qualitative data to achieve an initial model of the presentation (lost emotional self)[17] and potential necessary elements for therapeutic change (e.g. Drinkwater et al., in prep). This resulted in a clear hypothesised change process to be targeted in therapy[17]. SPEAKS

For peer review only - http://bmjopen.bmj.com/site/about/guidelines.xhtml

is organised into five phases, with associated mechanisms of change and therapeutic 'tasks' (see Figure 2 for brief overview). The intervention developers (AO, TL & HS) applied relevant psychological therapy models to target highlighted mechanisms, resulting in an integrative therapy drawing chiefly from emotion focused therapy (EFT) and schema therapy (ST). The therapy thus relies on dialogical self theory, and therapeutic tasks are those well established in EFT and ST, such as 'chairwork' interventions to enable 'parts of self' to communicate.

Intervention guidebook. The SPEAKS intervention is written up in a guidebook for therapists to follow. It outlines SPEAKS change process, mapping it onto expected 'phases' of therapy (Figure 2). In each phase, associated hypothesised mechanisms are outlined and 'therapeutic tasks' described. The guidebook meets requirements for preliminary feasibility evaluation as outlined in the stage model approach for developing a psychotherapy[21] and assists intervention adherence.

Therapist training & supervision. In additional to background training in an experiential dialogical self model, therapists receive four days SPEAKS model and therapy training, with further regular training days organised throughout the trial. Ongoing fortnightly supervision is delivered by SPEAKS developers. Where participants consent, all therapy sessions are video recorded for supervision. Regular reflective practice groups are offered by a SPEAKS developer and psychologist external to the clinical services (TL).

Treatment fidelity, untoward events and protocol adherence

Reviewing videotaped sessions in supervision ensures competent treatment delivery adhering to the SPEAKS model. This is usual good practice in both EFT and ST supervision models. Case records of each therapy session in client notes outline the phase of therapy, emotions focused upon, and therapeutic tasks employed.

For peer review only - http://bmjopen.bmj.com/site/about/guidelines.xhtml

Usual service SOPs regarding clinical care and risk management will be followed. Any protocol violations impacting delivery of SPEAKS such as admission to hospital will be recorded as per Trust guidance, and also logged in trial records for later reporting. Participants who are admitted to hospital will be withdrawn from the study.

Outcomes

Measures of acceptability. Triangulation of qualitative and quantitative data will address acceptability of SPEAKS to participants and therapists.

Qualitative. Participant and therapist lived experience of SPEAKS will be examined using post-intervention semi-structured interviews adapted from the Client Change Interview[22], completed by the researcher. Acceptability of aspects of the current and future trial designs are also addressed including use of questionnaires and willingness to be randomised to treatment arms.

Quantitative. Acceptability and perceived value of core SPEAKS components will be quantitatively assessed using visual analogue scales created for the study using questions based on the acceptability interview, and rated on a seven-point scale strongly agree to strongly disagree (e.g. I think the focus of SPEAKS makes sense for me and my difficulties). Numbers of people who choose to end their therapy because they do not think it is acceptable will also be calculated.

Measures of reach and recruitment. Screening of potential participants will be logged, and details of unmet inclusion criteria anonymously recorded. Recruitment yield will be monitored. Where participation is declined, reasons will be anonymously recorded. Completeness of measures at each time-point will be reported.

Measures of adherence and compliance. Treatment fidelity strategies will be employed consistent with the treatment fidelity checklist[23], including a clear intervention description (guide-book) and standardised therapist training. As described, session recordings will enable assessment of

For peer review only - http://bmjopen.bmj.com/site/about/guidelines.xhtml

adherence to SPEAKS, where consented to. A random selection of therapy tapes will be assessed for fidelity and adherence to the model judged by the intervention developers. They will assess core components of the treatments such as empathy (using the Therapist Empathy Scale [24]), appropriate task selection based on identified tasks markers, and appropriate task resolution. Data regarding treatment length, session numbers and content will be reported.

Measures for sample size estimation.

Clinical outcomes measures. In line with DSM V criteria for AN and OSFED-AN type (atypical AN) which emphasises rate of weight loss and ED cognitions, the primary outcome measure is of eating disorder cognitions and behaviours for sample size estimation - Eating Disorder Examination Questionnaire (EDEQ)[25]. Other indications of symptoms, such as BMI (kg/m^2); Depression, Anxiety and Stress Scales 21 (DASS-21)[26] and Clinical Impairment Assessment (CIA)[27] are also collected.

Intervention-specific measures. Other clinical and Intervention-specific measures will also be assessed as follows: Beliefs About Emotions Questionnaire[28], Young's Schema Questionnaire–Short form[29], Schema Mode Inventory ED–Short form (SMI-ED-SF)[30]; Silencing the Self Scale[31], The Sense of Agency Scale[32]; and Difficulties with Emotion Regulation Scale (DERS)[33].

Measures of economic evaluation. An adapted version of the Client Socio-demographic and Services Receipt Inventory (CSSRI)[34] will collect economic data pre- and post-intervention to assess treatment costs received in the six months preceding and during SPEAKS.

Measures of SPEAKS Change Process. The following analyses will test hypothesised therapeutic change process.

(1) Videoed therapy sessions will be analysed according to two coding systems to better understand change associated with better clinical outcomes, including hypotheses about both content and order of change. The Innovative Moments Coding System[35] is a

For peer review only - http://bmjopen.bmj.com/site/about/guidelines.xhtml

systematic reliable method for identifying innovative moments (IMs) in therapy, categorising by type (action, reflection, protest, reconceptualization, performing change). IMs are measured by the percentage of time spent elaborating on each IM (temporal salience). The Classification of Affective Meaning States (CAMS)[36] codes the presence of emotions expressed during therapy videos in one minute segments. The CAMS includes nine emotion categories: global distress (rejecting anger, fear/shame, negative self-evaluation, unmet need, relief, assertive anger/self-compassion, hurt/grief, and acceptance/agency) which will be analysed to assess changes in patterns of emotion expression over time including emotion types and frequency (cf. [37])

(2) Thematic analysis will be applied to psychological formulations of 'schema modes' constructed by participants during therapy as compared against those endorsed in a quantitative measure (SMI-ED[30]). This will enable us to improve the SPEAKS intervention based on the most salient schema modes, and mode changes associated with better outcomes.

Participants may participate without agreeing to their formulations or therapy recordings being analysed.

Participant timeline

Participants will be assessed using quantitative variables examining clinical and emotion change collected pre-intervention, at 3, 6 and 9 months into the intervention and post-intervention (12 months; Figure 3 and 4). Collecting questionnaire data every three months is standard practice within the Eating Disorder Services. Due to the coronavirus pandemic, some participants experienced a break in their SPEAKS therapy. In order to ensure that all participants are able to access the full 9-12 months of therapy intended, therapy will be extended for those impacted by a maximum of 3 months. Where relevant, this will be discussed and agreed between therapist and

For peer review only - http://bmjopen.bmj.com/site/about/guidelines.xhtml

participant. In order to capture end of therapy data for those affected, we will include an extra data collection time-point at 15 months just for these participants.

Qualitative acceptability interviews with service users will be completed at the end of each participant's involvement in the study (at 12 or 15 months follow-up). Therapist acceptability interviews will be completed when they have finished working with their final SPEAKS participant.

Sample size

The feasibility design must balance precision with unethical exposure of participants to the risks being monitored alongside unnecessary expense[38]. Teare and colleagues[39] recommend 35 participants for sufficient feasibility data and precision of mean and variance. Therapy attrition rates for people with AN can reach 40%[9]. With a sample size of 36, we will be able to estimate a drop-out rate of 40% to within a 95% confidence interval of +/- 16%. These data suggest an approximate revised sample size of up to 60 participants in order to achieve a sample of 36 participants completing therapy.

Recruitment

Patients will be consecutive referrals meeting inclusion/exclusion criteria to the two sites in South-East England: NELFT and SPFT. Participants will be identified from waiting lists in the first instance in order of referral. Recruitment will continue until SPEAKS therapists no longer have available spaces and will recommence when further spaces come available. Recruitment ended in February 2021. This was after journal submission, but prior to completion of peer reviews.

Methods: Data collection, management, and analysis

Allocation, allocation concealment and blinding

Not applicable due to the single arm design.

Data collection methods & retention

For peer review only - http://bmjopen.bmj.com/site/about/guidelines.xhtml

Informed consent and data at all time-points will be organised and collected by a research assistant specifically employed to complete this role. Mutually agreed appointment times for completion of all measures will be agreed. The research assistant will maintain engagement of participants with regular newsletters and individual correspondence.

Data management & availability

Data entry errors will be checked by double entering 10% of data. Examining data for impossible values by looking at data ranges will test data quality. No post-treatment data will be released until the database is locked. Access to the final trial dataset will be available to the PIs and research assistant. The datasets generated and/or analysed during the current study will be included in the subsequent results publication. Anonymised participant level data available on request.

Data analysis

The data will be analysed using password protected NHS computers at NELFT. It will be analysed by PIs, the research worker and clinical psychology trainees as part of their doctoral theses. Thematic analysis of acceptability interviews will be completed before quantitative analysis of acceptability data to avoid bias. Dependent t-tests (or non-parametric equivalents) and effect sizes (Cohen's d) will be calculated for all outcomes measures. EDEQ change will be utilised in a power analysis to estimate sample size required for effectiveness trial. We will model for missing data of anybody who completed therapy.

Methods: Monitoring

Data Monitoring

A Research Steering Committee (RSG) comprised of the research team including CI and PIs, representatives from the sponsors, representatives from both EDS and PPI members (with history of anorexia and family members) oversees the SPEAKS research programme. It is chaired by an

For peer review only

experienced researcher independent from trial Trusts and sponsor. The RSG meets every six months for reporting and discussion. Additional meetings can be convened at short notice if required. The RSG chair is available for independent advice to the research team.

Patient and Public Involvement

PPI has been in place since the inception of the study before trial funding was sought. An initial Research Design Service PPI Grant covered costs of a consultation series to assess SPEAKS model and trial validity. This informed intervention development, study design, and the SPEAKS name. Following trial funding, feedback on key documents such as the plain English summary and participant information sheets (PIS) were obtained via contacts made in this initial consultation. Further, PPI input continues throughout the study via the RSG and in open feedback events at key stages of the study advertised via local charity and support group networks.

Adverse event reporting and harms

Protocol violations such as hospital admission are recorded as per Trust guidance, and logged in trial records for reporting. Participants admitted to hospital will be withdrawn from the study. All research staff are NIHR Good Clinical Practice (GCP) trained and follow these guidelines for safety reporting procedures. Due to the feasibility design, there are no interim analyses or stopping guidelines; however, therapists monitor participant safety with regular risk assessments and communicate to the research team. Events leading to participants or others experiencing potential or actual serious harm are recorded by the researcher and reported to the PI and sponsor within 24 hours of knowledge of the event. Decision on expectedness and relatedness to the study intervention will be taken, with further investigation as required. The PI and sponsor will monitor events in case a pattern emerges, taking action if necessary. Clinical risk will be managed within ED service guidelines. Following completion of trial participation, all usual clinical service and NHS Trust

For peer review only - http://bmjopen.bmj.com/site/about/guidelines.xhtml

standard operating procedures continue to apply with post-trial care continuing if clinically indicated. During the trial all usual Trust complaint procedures can be followed.

Auditing

Overall study conduct, and conduct at individual sites, will be monitored by the Sponsor Monitor at regular intervals. The Trial Master File (TMF) will be audited at site initiation, annually for the duration of the study and at study closure. This audit will follow the sponsor Standard Operating Procedures (SOP) and includes checks of: completeness and secure storage of TMF, study-wide approvals, study-wide safety and deviation/violation reporting, GCP compliance and performance of study against recruitment targets.

Individual research sites will be monitored after five participants have been recruited as part of site initiation, annually from site initiation date and at study closure at site. Monitoring will follow the sponsor SOP and include monitoring of the Investigator Site File (ISF) for completeness and secure storage, SDV checks, CRF checks, Serious Adverse Events and deviation/violation checks, GCP compliance and performance of site against recruitment targets.

Ethics and dissemination

Research ethics approval

This study has been reviewed in accordance with the guidelines for Canterbury Christ Church University research and has been approved by the London – Bromley Research Ethics Committee (NHS Rec Reference REC Ref: 19/LO/1530).

Protocol amendments

Any protocol amendments will be communicated to all relevant parties and research sites. Notice of intention to submit an amendment will be provided via email to the sponsor and the research governance representatives at all sites to offer an opportunity to discuss any queries prior to

For peer review only - http://bmjopen.bmj.com/site/about/guidelines.xhtml

submission and confirm support. Ethical approval will subsequently be sought via online submission following IRAS guidance. Once ethical approval has been granted for an amendment this will be disseminated to all parties and official registries (such as ISRCTN) will be notified.

Consent

Patients meeting inclusion criteria will be provided with a PIS by their assessing clinician if they are willing to hear more about the study. Informed consent will be obtained at a face to face meeting with a member of the research team. The meeting will take place at least 48 hours after the participant has been provided with the PIS, with additional time given as necessary.

At the consent appointment, the research worker will answer any initial questions from the potential participant. They will review the PIS, highlighting key aspects and checking the patient's understanding of what study involvement entails. The researcher will explicitly state and make clear that deciding not to participate will not affect the patient's care in any way, and that if they decide to take part they can change their mind at any time without affecting their current or future care. The research team (like all other clinical staff members) will be trained in the principles of mental capacity and will hold this in mind throughout. Obtaining consent will be a focus of research supervision.

Confidentiality

In line with usual clinical practice, all participant information will be kept confidential, except as governed by law (i.e. if there is a legal obligation on the researcher to disclose this information to authorities due to risk concerns).

Signed informed consent forms will be stored separately from completed questionnaires and interview transcripts, which will be notated with only a participant number and/or pseudonym. Signed informed consent forms will be kept in a locked cabinet on NELFT or SPFT Property as relevant. Completed questionnaire data will be entered into study databases in encrypted Trust

For peer review only

For peer review only - http://bmjopen.bmj.com/site/about/guidelines.xhtml

network folders accessible only to the research team. Any video recordings of therapy sessions and formulation descriptions will be stored on secure encrypted password protected NHS hard drives.

Participant identity will not be included in written interview transcripts, and will not be revealed in any publication resulting from this study. Data gathered from this study will be retained as required by regulations, which is up to ten years following publication of empirical articles or communications describing study results.

Availability of data and materials

Study databases and TMFs will only be available to the research team employed by the participating Trusts. The trainee psychologists analysing video recordings and psychological formulation data will not know participant identity beyond what they see in recorded therapy sessions. Qualitative interview recordings may be transcribed by a third-party agency approved by the sponsor. Participant names and personal details will not be disclosed and full confidentiality agreements will be established as per this protocol.

Dissemination policy

Data will be disseminated via high-impact, peer-reviewed journals, with Open Access sought where possible. Papers will be submitted for conference presentations to achieve dissemination to practitioners and professionals within the ED field. Dissemination to service users and clinical networks is considered extremely important and be achieved via service user networks across NHS Trusts and HEIs involved in the study, via charity networks (e.g. UK eating disorder charity BEAT) and through a free open conference hosted by the NHS Trusts and HEI to disseminate findings and facilitate feedback on the SPEAKS clinical model and intervention. The decisions of when and where to publish data and authorship eligibility is decided by the SPEAKS RSG. Professional writers will not be used.

For peer review only - http://bmjopen.bmj.com/site/about/guidelines.xhtml

Discussion

This SPEAKS feasibility trial aims to assess acceptability, research and recruitment, sample size estimation and initial economic indications of the SPEAKS intervention for adults with AN Otherwise Specified Feeding or Eating Disorder-AN type. It also tests the hypothesised change process of the SPEAKS intervention.

Potential implications

This first test of the SPEAKS intervention will provide initial indication of the feasibility of SPEAKS and inform whether progression to larger trials is appropriate. Change process analyses will provide valuable insights into SPEAKS hypotheses, informing refinement of the intervention, whilst also contributing to broader evidence-base of relevant change processes for adults with AN.

Strengths

This feasibility trial is strengthened by multi-site design. Furthermore, sites included are relatively research naïve in delivery of clinical trials facilitating realistic insights into the feasibility of intervention delivery within naturalistic NHS services. Inclusion of consecutive referrals enables broad spectrum of participants across AN and OSFED severity and presentations, increasing generalisability. Robust measures to facilitate adherence to the model are included.

Challenges

SPEAKS is an experiential, relational model, designed for face-to-face settings. This trial began in the wake of the COVID-19 pandemic resulting in therapy sessions moving indefinitely to online video platforms with most participants never meeting their therapist in person. People with AN are often regarded as difficult to engage and have high levels of therapy dropout, thus issues of engagement

For peer review only

with clinical and research protocols are a challenge even without this context. These factors may affect outcomes or length of delivery.

For peer review only

For peer review only - http://bmjopen.bmj.com/site/about/guidelines.xhtml

Declaration of competing interests

Not applicable. The authors confirm there are no interests to declare.

Funding

This feasibility study is funded as part of an Integrated Clinical Academic Fellowship-Clinical Lectureship awarded to AO (ICA-CL-2015-01-005) supported by the National Institute for Health Research and Health Education England. The views expressed in this publication are those of the authors and not necessarily those of the NHS, the National Institute for Health Research, Health Education England or the Department of Health.

Roles and Responsibilities

Author affiliation & Contributions

AO is affiliated with Kent and Medway All Age Eating Disorder Service, North East London NHS Foundation Trust (NELFT), The Courtyard, Pudding Lane, Maidstone, ME14 1PA, United Kingdom and The Salomons Institute for Applied Psychology, Canterbury Christ Church University, Canterbury, United Kingdom. Email: anna.oldershaw@nelft.nhs.uk Tel: 0300 300 1980

AO is the Chief Investigator and Corresponding Author. She led protocol development and wrote all trial documents. She is co-developer of the SPEAKS intervention, and wrote the SPEAKS intervention guidebook. She is Principal Investigator (PI) for the Kent site.

TL is affiliated with The Salomons Institute for Applied Psychology, Canterbury Christ Church University, Canterbury, United Kingdom. Tony.Lavender@canterbury.ac.uk

TL is co-developer of the SPEAKS intervention and offered feedback and edits on the SPEAKS intervention guidebook. He contributed to the study design and development of the protocol. He is chair of the RSG.

For peer review only - http://bmjopen.bmj.com/site/about/guidelines.xhtml

RB is affiliated with Kent and Medway All Age Eating Disorder Service NELFT.

Randeep.Basra@nelft.nhs.uk

RB is research assistant on the SPEAKS feasibility trial. He has provided input into the protocol and assisted in the preparation and content of this manuscript.

HS is affiliated with Sussex Eating Disorders Service and Research and Development Department, Sussex Partnership NHS Foundation Trust (SPFT), Sussex, United Kingdom.

Helen.Startup@sussexpartnership.nhs.uk HS is co-developer of the SPEAKS intervention and provided content, feedback and edits on the SPEAKS intervention guidebook. She contributed to the study design and development of the protocol. She is PI for the Sussex site.

All authors read and approved the final manuscript.

Data sharing statement

Data are available upon reasonable request.

List of Figures

Figure 1. SPEAKS emotion change process

Figure 2. SPEAKS Treatment Phases

Figure 3. SPEAKS spirit schedule

Figure 4. SPEAKS Consort Diagram

References

For peer review only - http://bmjopen.bmj.com/site/about/guidelines.xhtml

1. Smink FR, Van Hoeken D, Hoek HW. Epidemiology of eating disorders: incidence, prevalence and mortality rates. Current psychiatry reports. 2012 Aug;14(4):406-14.
2. PricewaterhouseCoopers. The costs of eating disorders: Social, health and economic impacts. 2015. https://www.b-eat.co.uk/assets/000/000/302/The_costs_of_eating_disorders_Final_original.pdf Accessed 10 Dec, 2020.
3. Viana MC, Gruber MJ, Shahly V, Alhamzawi A, Alonso J, Andrade LH, Angermeyer MC, Benjet C, Bruffaerts R, Caldas-de-Almeida JM, Girolamo GD. Family burden related to mental and physical disorders in the world: results from the WHO World Mental Health (WMH) surveys. Brazilian Journal of Psychiatry. 2013 Jun;35(2):115-25.
4. National Institute of Health and Care Excellence Guideline. Eating disorders: recognition and treatment. NG69. London: National Institute of Health and Care Excellence. 2017.
5. Carter FA, Jordan J, McIntosh VV, Luty SE, McKenzie JM, Frampton CM, Bulik CM, Joyce PR. The long-term efficacy of three psychotherapies for anorexia nervosa: A randomized, controlled trial. International Journal of eating disorders. 2011 Nov;44(7):647-54.
6. Fairburn CG, Bailey-Straebler S, Basden S, Doll HA, Jones R, Murphy R, O'Connor ME, Cooper Z. A transdiagnostic comparison of enhanced cognitive behaviour therapy (CBT-E) and interpersonal psychotherapy in the treatment of eating disorders. Behaviour research and therapy. 2015 Jul 1;70:64-71.
7. Schmidt, U., Oldershaw, A., Jichi, F., Sternheim, L., Startup, H., McIntosh, V., ... & Landau, S. (2012). Out-patient psychological therapies for adults with anorexia nervosa: randomised controlled trial. *The British Journal of Psychiatry*, *201*(5), 392-399.
8. Schmidt U, Magill N, Renwick B, Keyes A, Kenyon M, Dejong H, Lose A, Broadbent H, Loomes R, Yasin H, Watson C. The Maudsley Outpatient Study of Treatments for Anorexia Nervosa and Related Conditions (MOSAIC): Comparison of the Maudsley Model of Anorexia Nervosa Treatment for Adults (MANTRA) with specialist supportive clinical management (SSCM) in

For peer review only - http://bmjopen.bmj.com/site/about/guidelines.xhtml

outpatients with broadly defined anorexia nervosa: A randomized controlled trial. Journal of consulting and clinical psychology. 2015 Aug;83(4):796.

9. Zipfel S, Wild B, Groß G, Friederich HC, Teufel M, Schellberg D, Giel KE, de Zwaan M, Dinkel A, Herpertz S, Burgmer M. Focal psychodynamic therapy, cognitive behaviour therapy, and optimised treatment as usual in outpatients with anorexia nervosa (ANTOP study): randomised controlled trial. The Lancet. 2014 Jan 11;383(9912):127-37.
10. DeJong H, Broadbent H, Schmidt U. A systematic review of dropout from treatment in outpatients with anorexia nervosa. International Journal of Eating Disorders. 2012 Jul;45(5):635-47.
11. Bulik CM. The challenges of treating anorexia nervosa. Lancet (London, England). 2014 Jan 11;383(9912):105.
12. Startup H, Mountford V, Lavender A, Schmidt U. Cognitive behavioural case formulation in complex eating disorders. Case formulation in cognitive behaviour therapy: The treatment of challenging and complex cases. 2015 Jul 6:239-64.
13. Kass AE, Kolko RP, Wilfley DE. Psychological treatments for eating disorders. Current opinion in psychiatry. 2013 Nov;26(6):549.
14. Sala M, Heard A, Black EA. Emotion-focused treatments for anorexia nervosa: a systematic review of the literature. Eating and Weight Disorders-Studies on Anorexia, Bulimia and Obesity. 2016 Jun 1;21(2):147-64.
15. Oldershaw A, Lavender T, Schmidt U. Are socio-emotional and neurocognitive functioning predictors of therapeutic outcomes for adults with anorexia nervosa?. European Eating Disorders Review. 2018 Jul;26(4):346-59.
16. Kendler KS, Campbell J. Interventionist causal models in psychiatry: repositioning the mind-body problem. Psychological medicine. 2009 Jun 1;39(6):881.

For peer review only

17. Oldershaw A, Startup H, Lavender T. Anorexia Nervosa and a lost emotional self: A psychological formulation of the development, maintenance, and treatment of Anorexia Nervosa. Frontiers in Psychology. 2019 Mar 4;10:219.

18. Oldershaw A, Startup H. 18 Building the Healthy Adult in eating disorders. Creative Methods in Schema Therapy: Advances and Innovation in Clinical Practice. 2020 Jun 18.

19. Royal College of Psychiatrists, Royal College of Pathologists, Royal College of Physicians. *MARSIPAN: Management of Really Sick Patients with Anorexia Nervosa, 2nd edition* (College Report CR189). Royal College of Psychiatrists.

20. Craig P, Dieppe P, Macintyre S, Michie S, Nazareth I, Petticrew M. Developing and evaluating complex interventions: the new Medical Research Council guidance. Bmj. 2008 Sep 29;337.

21. Carroll KM, Nuro KF. One size cannot fit all: A stage model for psychotherapy manual development. Clinical Psychology: Science and Practice. 2002 Dec;9(4):396-406.

22. Elliott, R., & Rodgers, B. (2008). Client Change Interview Schedule (v5). University of Strathclyde, Glasgow.

23. Borelli B, Sepinwall D, Ernst D, Bellg AJ, Czajkowski S, Breger R, DeFrancesco C, Levesque C, Sharp DL, Ogedegbe G, Resnick B. A new tool to assess treatment fidelity and evaluation of treatment fidelity across 10 years of health behavior research. Journal of consulting and clinical psychology. 2005 Oct;73(5):852.

24. Decker SE, Nich C, Carroll KM, Martino S. Development of the therapist empathy scale. Behavioural and cognitive psychotherapy. 2014 May;42(3):339-54.

25. Fairburn CG, Beglin SJ. Eating disorder examination questionnaire. Cognitive behavior therapy and eating disorders. 2008 Sep:309-13.

26. Henry JD, Crawford JR. The short-form version of the Depression Anxiety Stress Scales (DASS-21): Construct validity and normative data in a large non-clinical sample. British journal of clinical psychology. 2005 Jun;44(2):227-39.

For peer review only - http://bmjopen.bmj.com/site/about/guidelines.xhtml

27. Bohn K, Fairburn CG. The clinical impairment assessment questionnaire (CIA). Cognitive behavioral therapy for eating disorders. 2008:1-3.

28. Manser R, Cooper M, Trefusis J. Beliefs about emotions as a metacognitive construct: Initial development of a self-report questionnaire measure and preliminary investigation in relation to emotion regulation. Clinical Psychology & Psychotherapy. 2012 May;19(3):235-46.

29. Young JE, Brown G. Young Schema Questionnaire-Short Form; Version 3. Psychological Assessment. 2005.

30. Simpson SG, Pietrabissa G, Rossi A, Seychell T, Manzoni GM, Munro C, Nesci JB, Castelnuovo G. Factorial structure and preliminary validation of the Schema Mode Inventory for Eating Disorders (SMI-Ed). Frontiers in psychology. 2018 Apr 24;9:600.

31. Jack DC. The silencing the self scale. Fairhaven Faculty Publications. 2017.

32. Tapal A, Oren E, Dar R, Eitam B. The sense of agency scale: A measure of consciously perceived control over one's mind, body, and the immediate environment. Frontiers in psychology. 2017 Sep 12;8:1552.

33. Gratz KL, Roemer L. Multidimensional assessment of emotion regulation and dysregulation: Development, factor structure, and initial validation of the difficulties in emotion regulation scale. Journal of psychopathology and behavioral assessment. 2004 Mar 1;26(1):41-54.

34. Chisholm D, Knapp MR, Knudsen HC, Amaddeo F, Gaite LU, Van Wijngaarden BO, EPSILON Study Group. Client socio-demographic and service receipt inventory–European version: development of an instrument for international research: EPSILON Study 5. The British Journal of Psychiatry. 2000 Jul;177(S39):s28-33.

35. Gonçalves MM, Ribeiro AP, Mendes I, Matos M, Santos A. Tracking novelties in psychotherapy process research: The innovative moments coding system. Psychotherapy research. 2011 Sep 1;21(5):497-509.

For peer review only

For peer review only - http://bmjopen.bmj.com/site/about/guidelines.xhtml

36. Pascual-Leone A, Greenberg LS. Classification of affective-meaning states. A. Pascual-Leone, Emotional processing in the therapeutic hour: Why “the only way out is through. 2005:289-367.

37. Choi BH, Pos AE, Magnusson MS. Emotional change process in resolving self-criticism during experiential treatment of depression. Psychotherapy Research. 2016 Jul 3;26(4):484-99.

38. Billingham SA, Whitehead AL, Julious SA. An audit of sample sizes for pilot and feasibility trials being undertaken in the United Kingdom registered in the United Kingdom Clinical Research Network database. BMC medical research methodology. 2013 Dec;13(1):1-6.

39. Teare MD, Dimairo M, Shephard N, Hayman A, Whitehead A, Walters SJ. Sample size requirements to estimate key design parameters from external pilot randomised controlled trials: a simulation study. Trials. 2014 Dec;15(1):1-3.

For peer review only

For peer review only - http://bmjopen.bmj.com/site/about/guidelines.xhtml

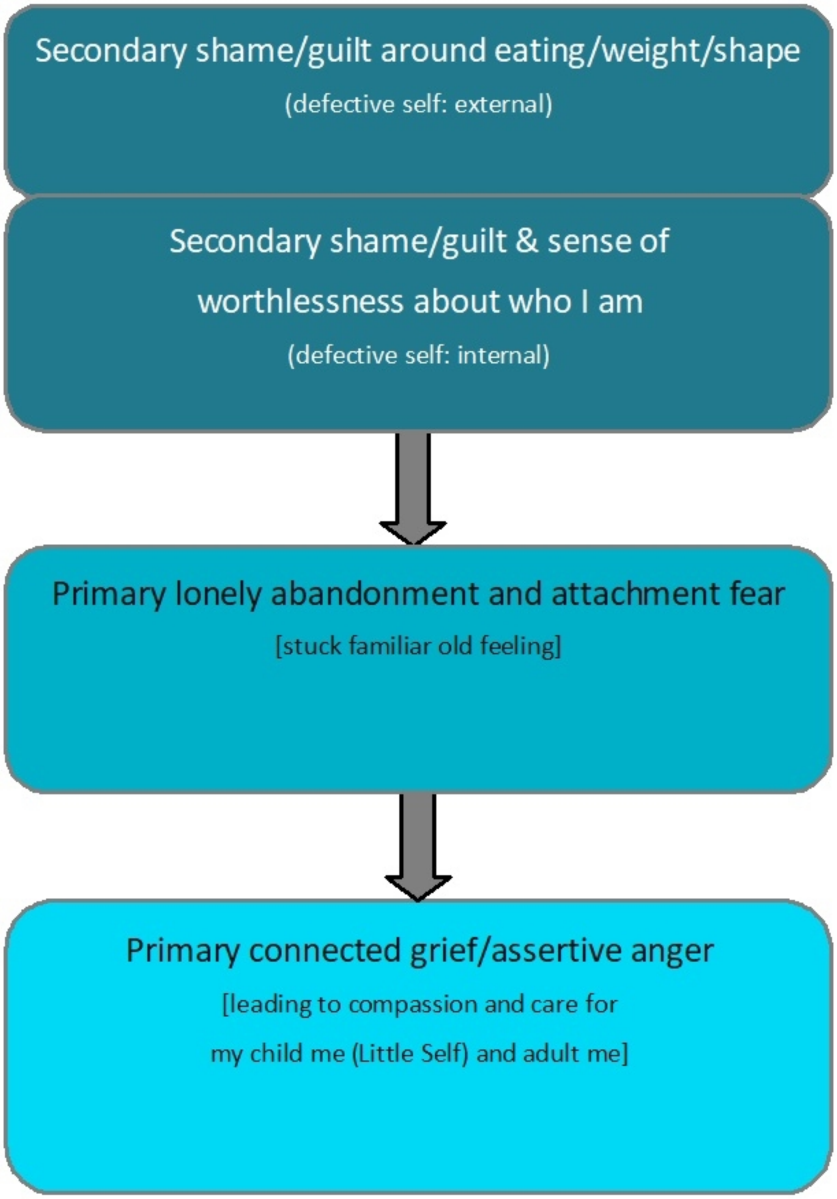

Figure 1. Emotion Change Process

129x185mm (120 x 120 DPI)

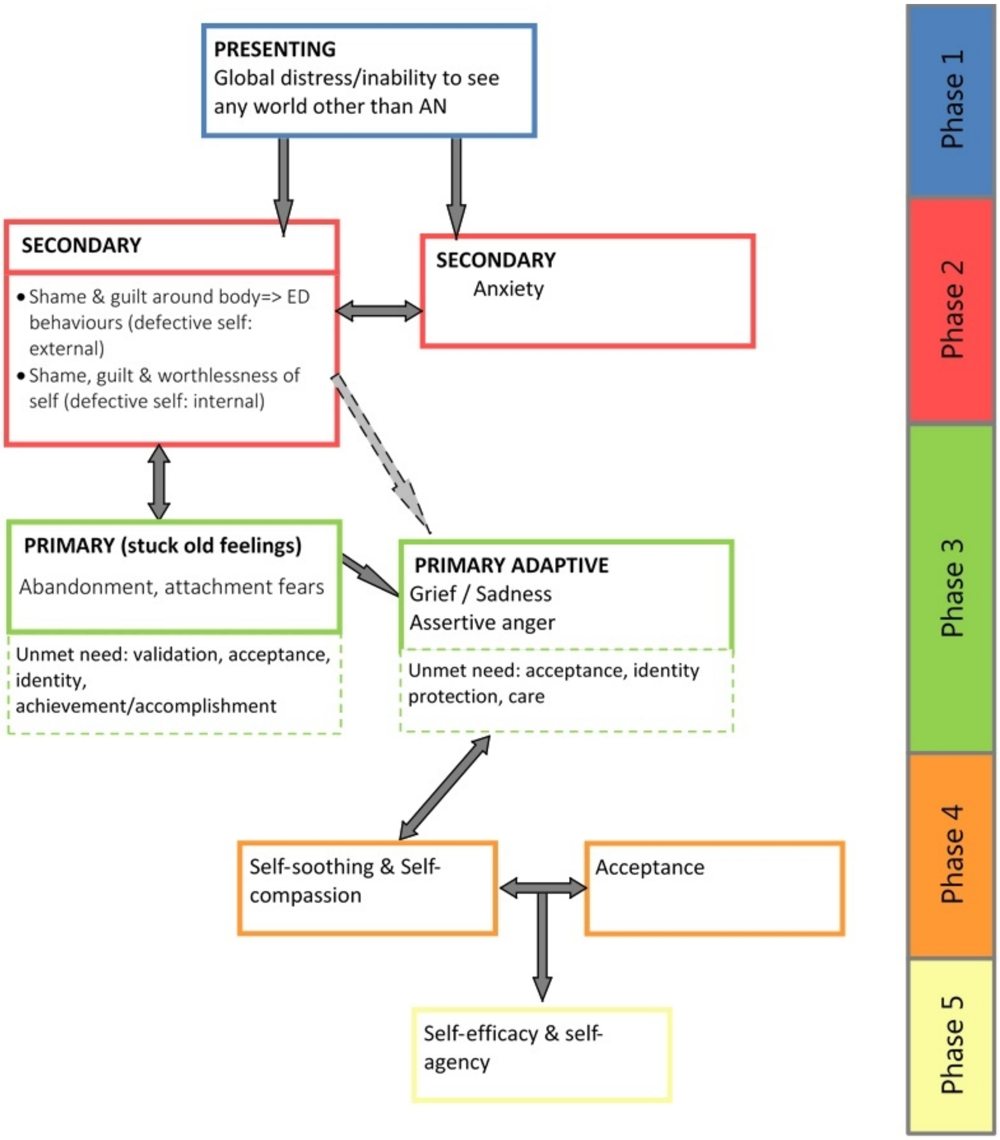

Figure 2. SPEAKS treatment phases

166x188mm (120 x 120 DPI)

		**SPEAKS STUDY PERIOD**				
	Enrolment	Post-enrolment				Post-intervention
TIMEPOINT	$-t_1$	Baseline	3 months	6 months	9 months	12-15 months
ENROLMENT:						
Eligibility screen	X					
Informed consent	X					
INTERVENTION:						
SPEAKS ASSESSMENTS						
Adapted CSSRI		X				X
Clinical outcome measures		X	X	X	X	X
Intervention-specific measures		X	X	X	X	X
Qualitative interviews						X

Figure 3. Spirit Schedule

188x176mm (120 x 120 DPI)

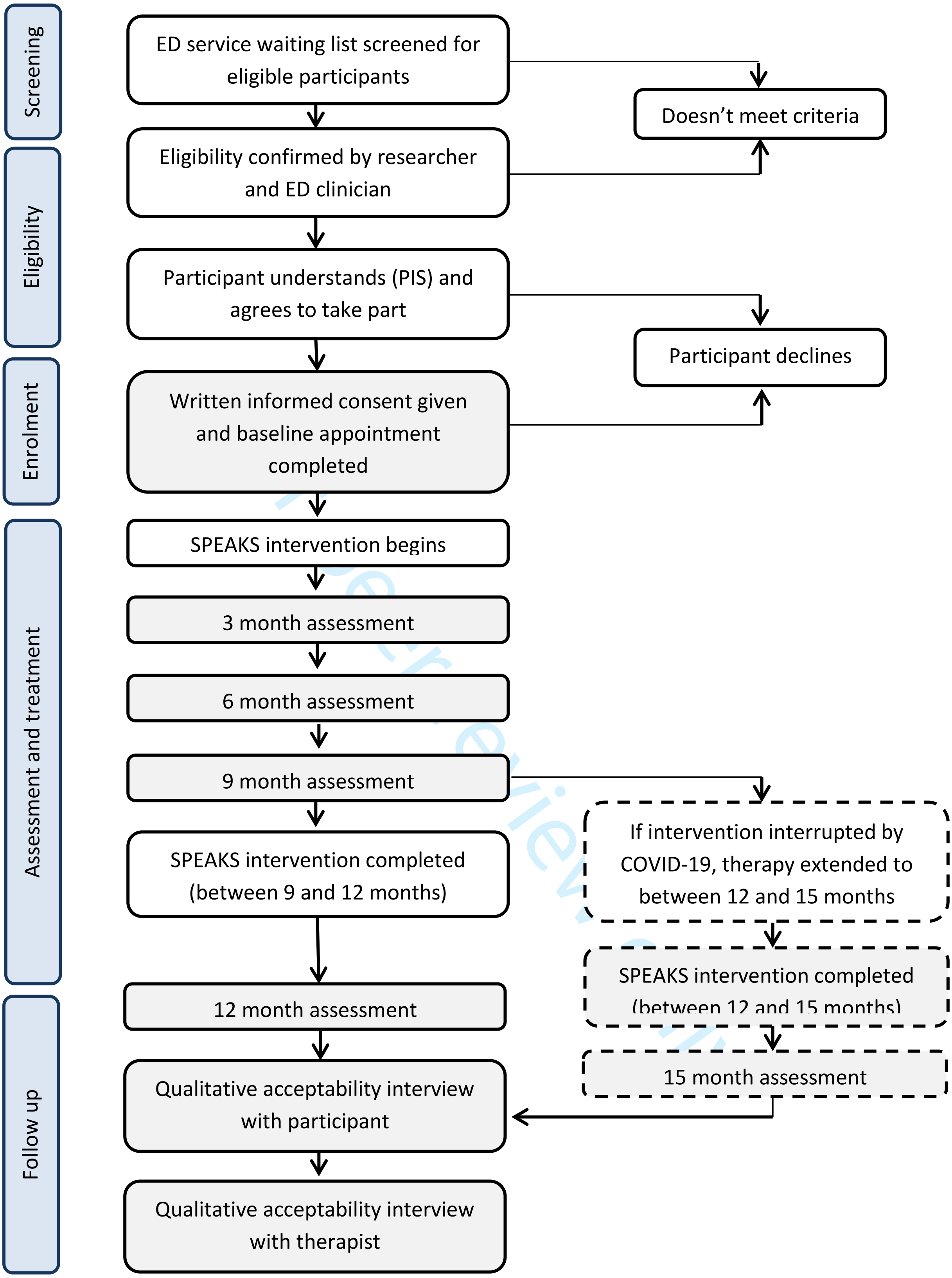
Screening
Eligibility
Enrolment
Assessment and treatment
Follow up
ED service waiting list screened for eligible participants
Doesn't meet criteria
Eligibility confirmed by researcher and ED clinician
Participant understands (PIS) and agrees to take part
Participant declines
Written informed consent given and baseline appointment completed
SPEAKS intervention begins
3 month assessment
6 month assessment
9 month assessment
If intervention interrupted by COVID-19, therapy extended to between 12 and 15 months
SPEAKS intervention completed (between 9 and 12 months)
SPEAKS intervention completed (between 12 and 15 months)
12 month assessment
15 month assessment
Qualitative acceptability interview with participant
Qualitative acceptability interview with therapist

SPIRIT

STANDARD PROTOCOL ITEMS: RECOMMENDATIONS FOR INTERVENTIONAL TRIALS

SPIRIT 2013 Checklist: Recommended items to address in a clinical trial protocol and related documents* – completed for SPEAKS Feasibility Trial protocol

Section/item	ItemNo	Description	Addressed on page(s)
**Administrative information**			
Title	1	Descriptive title identifying the study design, population, interventions, and, if applicable, trial acronym	1
Trial registration	2a	Trial identifier and registry name. If not yet registered, name of intended registry	4
	2b	All items from the World Health Organization Trial Registration Data Set	Available in trial registry
Protocol version	3	Date and version identifier	4
Funding	4	Sources and types of financial, material, and other support	22
Roles and responsibilities	5a	Names, affiliations, and roles of protocol contributors	22
	5b	Name and contact information for the trial sponsor	4
	5c	Role of study sponsor and funders, if any, in study design; collection, management, analysis, and interpretation of data; writing of the report; and the decision to submit the report for publication, including whether they will have ultimate authority over any of these activities	16-19
	5d	Composition, roles, and responsibilities of the coordinating centre, steering committee, endpoint adjudication committee, data management team, and other individuals or groups overseeing the trial, if applicable (see Item 21a for data monitoring committee)	16

For peer review only

**Introduction**			
Background and rationale	6a	Description of research question and justification for undertaking the trial, including summary of relevant studies (published and unpublished) examining benefits and harms for each intervention	5
	6b	Explanation for choice of comparators	n/a
Objectives	7	Specific objectives or hypotheses	6
Trial design	8	Description of trial design including type of trial (eg, parallel group, crossover, factorial, single group), allocation ratio, and framework (eg, superiority, equivalence, noninferiority, exploratory)	7
**Methods: Participants, interventions, and outcomes**			
Study setting	9	Description of study settings (eg, community clinic, academic hospital) and list of countries where data will be collected. Reference to where list of study sites can be obtained	8
Eligibility criteria	10	Inclusion and exclusion criteria for participants. If applicable, eligibility criteria for study centres and individuals who will perform the interventions (eg, surgeons, psychotherapists)	8
Interventions	11a	Interventions for each group with sufficient detail to allow replication, including how and when they will be administered	9
	11b	Criteria for discontinuing or modifying allocated interventions for a given trial participant (eg, drug dose change in response to harms, participant request, or improving/worsening disease)	9
	11c	Strategies to improve adherence to intervention protocols, and any procedures for monitoring adherence (eg, drug tablet return, laboratory tests)	10-12

For peer review only - http://bmjopen.bmj.com/site/about/guidelines.xhtml

	11d	Relevant concomitant care and interventions that are permitted or prohibited during the trial	9
Outcomes	12	Primary, secondary, and other outcomes, including the specific measurement variable (eg, systolic blood pressure), analysis metric (eg, change from baseline, final value, time to event), method of aggregation (eg, median, proportion), and time point for each outcome. Explanation of the clinical relevance of chosen efficacy and harm outcomes is strongly recommended	12-13
Participant timeline	13	Time schedule of enrolment, interventions (including any run-ins and washouts), assessments, and visits for participants. A schematic diagram is highly recommended (see Figure)	14
Sample size	14	Estimated number of participants needed to achieve study objectives and how it was determined, including clinical and statistical assumptions supporting any sample size calculations	14
Recruitment	15	Strategies for achieving adequate participant enrolment to reach target sample size	14
**Methods: Assignment of interventions (for controlled trials)**			
Allocation:			
Sequence generation	16a	Method of generating the allocation sequence (eg, computer-generated random numbers), and list of any factors for stratification. To reduce predictability of a random sequence, details of any planned restriction (eg, blocking) should be provided in a separate document that is unavailable to those who enrol participants or assign interventions	15

Allocation concealment mechanism	16b	Mechanism of implementing the allocation sequence (eg, central telephone; sequentially numbered, opaque, sealed envelopes), describing any steps to conceal the sequence until interventions are assigned	15
Implementation	16c	Who will generate the allocation sequence, who will enrol participants, and who will assign participants to interventions	15
Blinding (masking)	17a	Who will be blinded after assignment to interventions (eg, trial participants, care providers, outcome assessors, data analysts), and how	15
	17b	If blinded, circumstances under which unblinding is permissible, and procedure for revealing a participant's allocated intervention during the trial	n/a
**Methods: Data collection, management, and analysis**			
Data collection methods	18a	Plans for assessment and collection of outcome, baseline, and other trial data, including any related processes to promote data quality (eg, duplicate measurements, training of assessors) and a description of study instruments (eg, questionnaires, laboratory tests) along with their reliability and validity, if known. Reference to where data collection forms can be found, if not in the protocol	15
	18b	Plans to promote participant retention and complete follow-up, including list of any outcome data to be collected for participants who discontinue or deviate from intervention protocols	15
Data management	19	Plans for data entry, coding, security, and storage, including any related processes to promote data quality (eg, double data entry; range checks for data values). Reference to where details of data management procedures can be found, if not in the protocol	15

For peer review only - http://bmjopen.bmj.com/site/about/guidelines.xhtml

Statistical methods	20a	Statistical methods for analysing primary and secondary outcomes. Reference to where other details of the statistical analysis plan can be found, if not in the protocol	15-16
	20b	Methods for any additional analyses (eg, subgroup and adjusted analyses)	15-16
	20c	Definition of analysis population relating to protocol non-adherence (eg, as randomised analysis), and any statistical methods to handle missing data (eg, multiple imputation)	15-16
**Methods: Monitoring**			
Data monitoring	21a	Composition of data monitoring committee (DMC); summary of its role and reporting structure; statement of whether it is independent from the sponsor and competing interests; and reference to where further details about its charter can be found, if not in the protocol. Alternatively, an explanation of why a DMC is not needed	16
	21b	Description of any interim analyses and stopping guidelines, including who will have access to these interim results and make the final decision to terminate the trial	16
Harms	22	Plans for collecting, assessing, reporting, and managing solicited and spontaneously reported adverse events and other unintended effects of trial interventions or trial conduct	16-17
Auditing	23	Frequency and procedures for auditing trial conduct, if any, and whether the process will be independent from investigators and the sponsor	17
**Ethics and dissemination**			
Research ethics approval	24	Plans for seeking research ethics committee/institutional review board (REC/IRB) approval	18

For peer review only - http://bmjopen.bmj.com/site/about/guidelines.xhtml

Protocol amendments	25	Plans for communicating important protocol modifications (eg, changes to eligibility criteria, outcomes, analyses) to relevant parties (eg, investigators, REC/IRBs, trial participants, trial registries, journals, regulators)	18
Consent or assent	26a	Who will obtain informed consent or assent from potential trial participants or authorised surrogates, and how (see Item 32)	18
	26b	Additional consent provisions for collection and use of participant data and biological specimens in ancillary studies, if applicable	n/a
Confidentiality	27	How personal information about potential and enrolled participants will be collected, shared, and maintained in order to protect confidentiality before, during, and after the trial	19
Declaration of interests	28	Financial and other competing interests for principal investigators for the overall trial and each study site	22
Access to data	29	Statement of who will have access to the final trial dataset, and disclosure of contractual agreements that limit such access for investigators	15
Ancillary and post-trial care	30	Provisions, if any, for ancillary and post-trial care, and for compensation to those who suffer harm from trial participation	17
Dissemination policy	31a	Plans for investigators and sponsor to communicate trial results to participants, healthcare professionals, the public, and other relevant groups (eg, via publication, reporting in results databases, or other data sharing arrangements), including any publication restrictions	20
	31b	Authorship eligibility guidelines and any intended use of professional writers	20

For peer review only

	31c	Plans, if any, for granting public access to the full protocol, participant-level dataset, and statistical code	15
**Appendices**			
Informed consent materials	32	Model consent form and other related documentation given to participants and authorised surrogates	Appendix
Biological specimens	33	Plans for collection, laboratory evaluation, and storage of biological specimens for genetic or molecular analysis in the current trial and for future use in ancillary studies, if applicable	n/a

*It is strongly recommended that this checklist be read in conjunction with the SPIRIT 2013 Explanation & Elaboration for important clarification on the items. Amendments to the protocol should be tracked and dated. The SPIRIT checklist is copyrighted by the SPIRIT Group under the Creative Commons "Attribution-NonCommercial-NoDerivs 3.0 Unported" license.

For peer review only

TIDieR
Template for Intervention Description and Replication

The TIDieR (Template for Intervention Description and Replication) Checklist*:

Information to include when describing an intervention and the location of the information

Item number	Item	Where located ** Primary paper (page or appendix number)	Other † (details)
	**BRIEF NAME**		
**1.**	Provide the name or a phrase that describes the intervention.	Title page 1	
	**WHY**		
**2.**	Describe any rationale, theory, or goal of the elements essential to the intervention.	Intro page 5	
	**WHAT**		
**3.**	Materials: Describe any physical or informational materials used in the intervention, including those provided to participants or used in intervention delivery or in training of intervention providers. Provide information on where the materials can be accessed (e.g. online appendix, URL).	page 10	
**4.**	Procedures: Describe each of the procedures, activities, and/or processes used in the intervention, including any enabling or support activities.	page 10	
	**WHO PROVIDED**		
**5.**	For each category of intervention provider (e.g. psychologist, nursing assistant), describe their expertise, background and any specific training given.	page 9	
	**HOW**		
**6.**	Describe the modes of delivery (e.g. face-to-face or by some other mechanism, such as internet or telephone) of the intervention and whether it was provided individually or in a group.	page 9	
	**WHERE**		
**7.**	Describe the type(s) of location(s) where the intervention occurred, including any necessary infrastructure or relevant features.	page 9	

For peer review only - http://bmjopen.bmj.com/site/about/guidelines.xhtml

	**WHEN and HOW MUCH**		
**8.**	Describe the number of times the intervention was delivered and over what period of time including the number of sessions, their schedule, and their duration, intensity or dose.	page 9	
	**TAILORING**		
**9.**	If the intervention was planned to be personalised, titrated or adapted, then describe what, why, when, and how.	page 9	
	**MODIFICATIONS**		
**10.‡**	If the intervention was modified during the course of the study, describe the changes (what, why, when, and how).	n/a	
	**HOW WELL**		
**11.**	Planned: If intervention adherence or fidelity was assessed, describe how and by whom, and if any strategies were used to maintain or improve fidelity, describe them.	page 11-12	
**12.‡**	Actual: If intervention adherence or fidelity was assessed, describe the extent to which the intervention was delivered as planned.	n/a	

** **Authors** - use N/A if an item is not applicable for the intervention being described. **Reviewers** – use '?' if information about the element is not reported/not sufficiently reported.

† If the information is not provided in the primary paper, give details of where this information is available. This may include locations such as a published protocol or other published papers (provide citation details) or a website (provide the URL).

‡ If completing the TIDieR checklist for a protocol, these items are not relevant to the protocol and cannot be described until the study is complete.

* We strongly recommend using this checklist in conjunction with the TIDieR guide (see *BMJ* 2014;348:g1687) which contains an explanation and elaboration for each item.

* The focus of TIDieR is on reporting details of the intervention elements (and where relevant, comparison elements) of a study. Other elements and methodological features of studies are covered by other reporting statements and checklists and have not been duplicated as part of the TIDieR checklist. When a **randomised trial** is being reported, the TIDieR checklist should be used in conjunction with the CONSORT statement (see www.consort-statement.org) as an extension of **Item 5 of the CONSORT 2010 Statement.** When a **clinical trial protocol** is being reported, the TIDieR checklist should be used in conjunction with the SPIRIT statement as an extension of **Item 11 of the SPIRIT 2013 Statement** (see www.spirit-statement.org). For alternate study designs, TIDieR can be used in conjunction with the appropriate checklist for that study design (see www.equator-network.org).

For peer review only - http://bmjopen.bmj.com/site/about/guidelines.xhtml
